# Global Patterns of Bacterial Beta-Diversity in Seafloor and Seawater Ecosystems

**DOI:** 10.1371/journal.pone.0024570

**Published:** 2011-09-08

**Authors:** Lucie Zinger, Linda A. Amaral-Zettler, Jed A. Fuhrman, M. Claire Horner-Devine, Susan M. Huse, David B. Mark Welch, Jennifer B. H. Martiny, Mitchell Sogin, Antje Boetius, Alban Ramette

**Affiliations:** 1 Microbial Habitat Group, Max Planck Institute for Marine Microbiology, Bremen, Germany; 2 Josephine Bay Paul Center, Marine Biological Laboratory, Woods Hole, Massachusetts, United States of America; 3 Department of Biological Sciences, University of Southern California, Los Angeles, California, United States of America; 4 School of Aquatic and Fishery Sciences, University of Washington, Seattle, Washington, United States of America; 5 Department of Ecology and Evolutionary Biology, University of California Irvine, Irvine, California, United States of America; 6 HGF MPG Joint Research Group on Deep Sea Ecology and Technology, Alfred Wegener Institute for Polar and Marine Research, Bremerhaven, Germany; Argonne National Laboratory, United States of America

## Abstract

**Background:**

Marine microbial communities have been essential contributors to global biomass, nutrient cycling, and biodiversity since the early history of Earth, but so far their community distribution patterns remain unknown in most marine ecosystems.

**Methodology/Principal Findings:**

The synthesis of 9.6 million bacterial V6-rRNA amplicons for 509 samples that span the global ocean's surface to the deep-sea floor shows that pelagic and benthic communities greatly differ, at all taxonomic levels, and share <10% bacterial types defined at 3% sequence similarity level. Surface and deep water, coastal and open ocean, and anoxic and oxic ecosystems host distinct communities that reflect productivity, land influences and other environmental constraints such as oxygen availability. The high variability of bacterial community composition specific to vent and coastal ecosystems reflects the heterogeneity and dynamic nature of these habitats. Both pelagic and benthic bacterial community distributions correlate with surface water productivity, reflecting the coupling between both realms by particle export. Also, differences in physical mixing may play a fundamental role in the distribution patterns of marine bacteria, as benthic communities showed a higher dissimilarity with increasing distance than pelagic communities.

**Conclusions/Significance:**

This first synthesis of global bacterial distribution across different ecosystems of the World's oceans shows remarkable horizontal and vertical large-scale patterns in bacterial communities. This opens interesting perspectives for the definition of biogeographical biomes for bacteria of ocean waters and the seabed.

## Introduction

Microbes are essential to the ocean in terms of biomass, diversity [1], [2], [3] and ecosystem functioning [4], [5]. Understanding patterns of microbial distribution is therefore crucial if we are to anticipate the responses of marine ecosystems to future environmental changes. The ocean is the largest contiguous environment on Earth, but it displays a basic subdivision into the pelagic (i.e. water column) and the benthic (i.e. sediment) realms. Both realms differ profoundly in terms of physical, chemical, and biological properties, as well as spatial and temporal scales of variability [3], [6]. Seawater is characterised by strong physical mixing due to currents and storms, a variable nutrient state, and the occurrence of widely distributed, diluted microbes [1], [4]. In contrast, most of the seafloor realm consists of less dynamic environments and offers a vast matrix of inorganic and organic solid surfaces with heterogeneous and complex organic polymers as substrates for bacterial growth [2], [7]. Although fundamentally different in physical and chemical properties, the seawater and seafloor realms are connected through the sedimentation of organic matter produced in and sinking from the euphotic zone [8], [9].

Patterns of beta-diversity of microbial communities, i.e. how microbial assemblages vary in space and along environmental gradients [10], have been of long-standing interest in the field of marine microbiology. From the ocean surface to the deep seafloor, the decreasing light penetration, temperature and availability of labile organic matter with increasing water depth have been identified as important factors that determine the vertical distribution and stratification of microbial communities [1], [11], [12]. Regarding the horizontal patterns of marine bacterial community composition, it is now well established that the Candidatus *SAR11* and *Prochlorococcus* clades dominate surface waters globally [1], [13], [14]. A few investigations have also reported distinct bacterial communities in different ocean water masses [15], [16], similar to phyto- and zooplankton [17], [18]. Other studies have also shown the existence of biogeographical patterns of individual bacterial taxa [19] or communities that correlate with habitat type and climate [1], [13], [20], [21], [22]. However, most of these studies have focused on the photosynthetically productive euphotic zone, which accounts for less than 10% of the total volume of water in the ocean, and global surveys of microbial diversity in seafloor ecosystems are still missing. Although community composition and distribution of macroorganisms differ strongly between benthic and pelagic environments [23], one may yet expect a different picture for marine microbes because seafloor sediments are composed of particles sinking from overlying ocean waters, which may result in certain similarities between pelagic and benthic microbial communities [24], [25]. It is, however, not what has been found so far in studies performed at the local scale based on different approaches, which overall tend to support the idea that pelagic and benthic microbial community composition differ (reviewed in [26]). This hypothesis still needs to be verified at the global scale by using a consistent technical approach, and environmental and spatial factors responsible for changes in microbial community composition (*i.e.* beta-diversity) need to be determined.

According to the Baas-Becking and Beijerink hypothesis “everything is everywhere, but, the environment selects” [27], unlimited dispersal and abiotic environmental filtering are responsible for the different distributions of microbial populations on Earth. Recent observations, however, have nuanced this principle by presenting evidence of both cosmopolitanism (i.e. global occurrence) [28], and conversely, provincialism (i.e. geographically localised occurrence) for some microbial species [29], [30]. These contradicting patterns still provoke debates in microbial ecology, but might actually arise from differences in (i) the spatial scales and taxonomic resolutions at which studies have been conducted [29], [30], [31], [32] and (ii) The ecosystem types considered for the description of microbial biogeographical patterns [23]. Concerning the first point, assessing the taxonomic depth at which ecological signatures are detectable and the consistency of these signatures across taxonomic ranks, are fundamental and current questions being addressed in the ecology and evolution of both “macro”-bial and microbial communities [31], [33], [34]. Hence, the use of large, global datasets encompassing diverse habitats in the pelagic and benthic realms is an essential next step in marine microbial biogeography.

**Figure 1:**
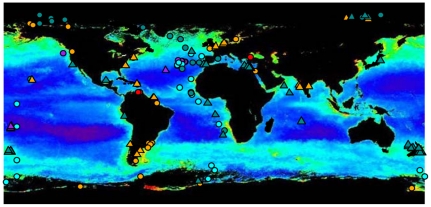
Global map of sample locations. Sample locations were plotted on a SeaWiFS satellite image of chlorophyll pigment concentration, with redder colour representing higher concentrations. The realm origin of the samples is indicated by circles (pelagic) and triangles (benthic), whereas ecosystem types are indicated by the colour orange (coastal), light blue (open ocean surface waters), dark blue (deep-sea), red (anoxic), and purple (hydrothermal vents). Further visualization of sample distribution e.g. on a bathymetric map is available at http://vamps.mbl.edu/mapper/index.php.

Here we provide a first global synthesis of bacterial sequences obtained from the open access dataset of the International Census of Marine Microbes (ICoMM) [35], in order to compare bacterial beta-diversity for the benthic and pelagic realms and some of their key ecosystems (Fig. 1, Table 1). This dataset includes 509 globally distributed samples from 37 individual projects [35] (Fig. 1). The 9.6 million rRNA sequences of ribosomal V6-rRNA pyrotag amplicons obtained by 454 pyrosequencing were used in combination with a set of broad proxies which categorize complex physical, chemical and biological characteristics of ocean ecosystems, such as distance to coast, water depth, and different productivity indices. Our results show unexpected biogeographical patterns of bacterial communities, which reflect physical, chemical and biological contrasts, but also biogeochemical interconnections between the pelagic and the benthic realms.

**Table 1:** Sample characteristics by realm and ecosystem type.

Realm	Ecosystem type§	Number of samples	Number of sequences	Number of OTU_0.03_ $	Number of OTU_0.03_ singletons$
Pelagic					
	Coastal	194	2,561,067	20,364	10,770
	Surface Open Ocean	70	1,570,433	8,995	4,223
	Deep Open Ocean	61	1,161,147	10,846	4,940
	Vents*	14	911,066	15,072	7,290
	Anoxic*	17	262,995	3,759	1,477
	Total	356	6,466,708	44,493	23,103
Benthic					
	Coastal	72	1,624,189	59,051	27,648
	Deep seafloor	68	1,323,228	40,680	18,544
	Vents*	13	173,725	2,885	1,169
	Total	153	3,121,142	89,168	41,831
					
Grand Total:		509	9,587,850	120,436	59,913

§ Coastal samples were located at less than 200 nautical miles from the shoreline and less than 200 m water depth. Surface Open Ocean refers to offshore samples from the top 200 m of the water column, whereas Deep Open Ocean refers to offshore samples below 200 m water depth. Deep Seafloor refers to sediment samples that were located below 200 m water depth. Vents and Anoxic Ecosystems were identified from project descriptions, available on MICROBIS (See Material and Methods).

$ OTU_0.03:_ Operational Taxonomic Unit at 3% of sequence dissimilarity. These numbers are only indicative and are not equivalent to bacterial community richness (See Material and Methods).

*These samples were not kept for variation partitioning analyses due to insufficient number of samples.

## Results

### Dataset description

The ICoMM dataset comprises samples from a wide range of ocean ecosystem types which we clustered according to their classical distinction in oceanography such as surface and deep water, coasts and open ocean, pelagic and benthic (see Material and Methods; Table 1). Additionally, we compared ICoMM samples collected from hydrothermal vents and anoxic waters. Our analysis did not include samples from animal microbiomes (e.g. ICoMM sponge or coral samples) because they represented a very small number of collection sites. The ICoMM projects whose data have been analysed here employed identical PCR primers, amplification, pyrosequencing and data cleaning and annotation protocols for the very same region of ribosomal RNA genes, which allow for a standardized comparison of the bacterial communities at the global scale. Using 509 selected environmental samples (Fig. 1), the sequence trimming and processing (see Material and Methods) resulted in a total of 9,587,850 DNA sequences related to Bacteria, which clustered into 120,436 Operational Taxonomic Units at 3% sequence dissimilarity level (OTU_0.03_, Table 1). The large number of OTUs observed in the total dataset represents sequences with an average error rate estimated to be 1/400 positions [36]. We used the SLP clustering strategy that minimizes inflation of the number of clusters (OTUs) and hence limits overestimation of diversity [37]. Given the undersampling of global bacterial diversity in public databases, taxonomic annotation automatically excluded an increasing proportion of environmental sequences with increasing taxonomic resolution (Table S1). More particularly, the percentages of taxonomically assigned OTUs_0.03_ in pelagic or benthic samples varied between 16 and 23% from the phylum to the genus level, with the exception of the order level for which only 8.5% of the OTUs were identified. The sequencing effort per sample was similar for pelagic and benthic samples (Table 1). Globally and in each ecosystem type, ∼50% of the OTU_0.03_ were singletons, i.e. occurred only once in the full dataset. However, these singletons only accounted for ∼0.6% of all sequences obtained (Table 1). OTU_0.03_ number was much higher in the benthic than in the pelagic realm, despite a smaller number of samples. Bacterial communities were found to be significantly less even in the pelagic than in the benthic realm (Simpson's Inverse Index of Diversity  = 20.2±9.30 and 143.2±111.54 for pelagic and benthic samples, respectively; Mann-Whitney rank sum test *P*<0.001). In this first synthesis approach our main questions were as to the composition, structure and similarity of bacterial communities across different ocean realms and ecosystems. Other richness estimates of pelagic and benthic communities sampled by ICoMM can be obtained via VAMPS (http://vamps.mbl.edu/diversity/diversity.php).

### Description of the main taxa per realm and ecosystem type

**Figure 2:**
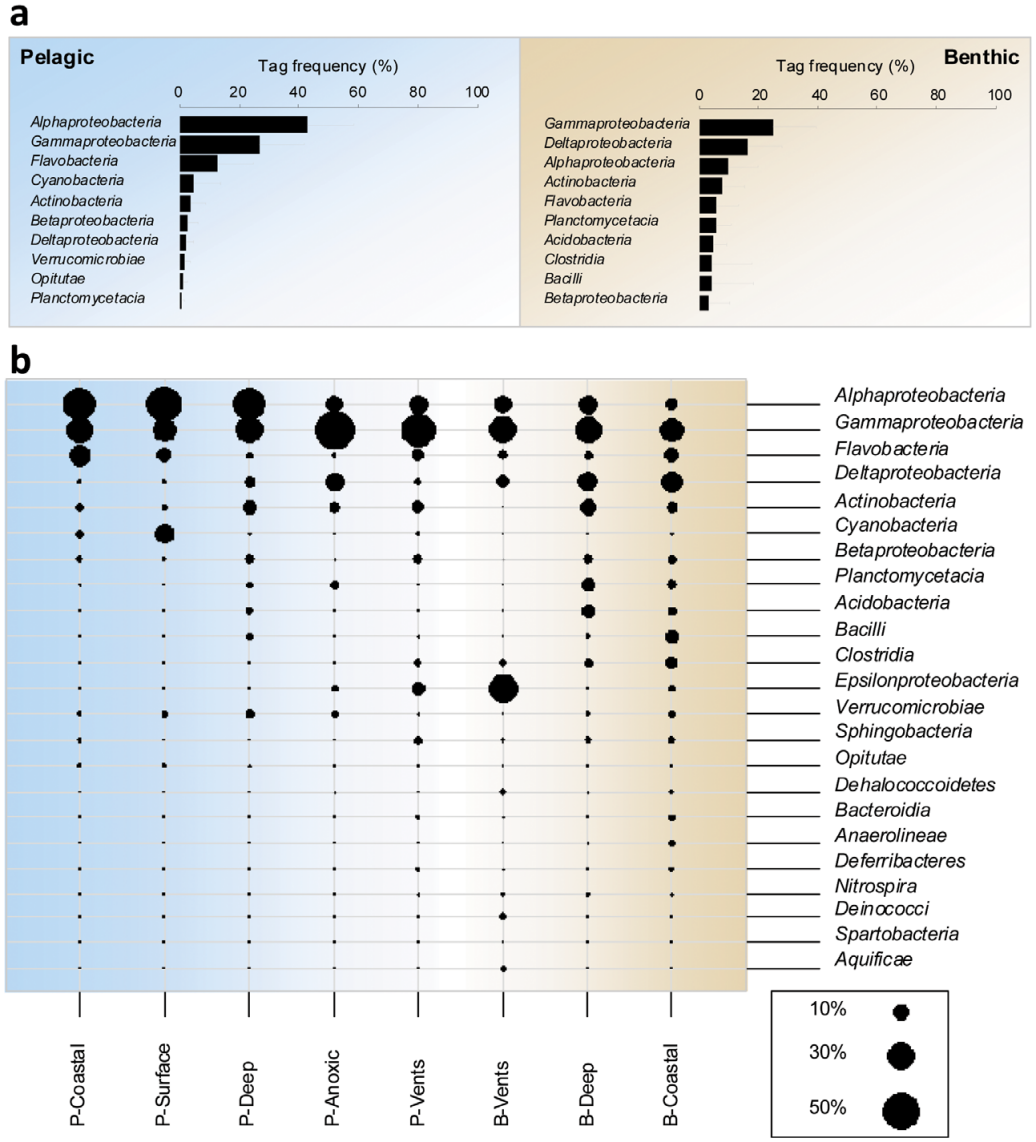
Bacterial community composition according to realms and ecosystem types. (**a**) Average sequence frequency for the ten most abundant bacterial classes in the pelagic and benthic realms. Error bars represent standard deviation values (number of samples indicated in Table 1). Vents and anoxic ecosystems were not taken into account for the average bacterial community composition in pelagic and benthic realms. (**b**) Average proportions of the main bacterial taxa per realm and ecosystem type. P =  Pelagic, B =  Benthic. Notice that taxonomic levels displayed here are not necessarily of the same level, but reflect the most common levels whose ecology and diversity are usually investigated in marine microbiology.

We assessed the relative sequence abundance of the major bacterial taxa at the Class level for each realm (excluding vents and anoxic ecosystems due to their much smaller sample size; Fig. 2a). Sequences of *Gammaproteobacteria* generally dominated in both pelagic and benthic samples. *Alphaproteobacteria,* mainly represented by the *SAR11* cluster (37±21.2% of the total sequences identified at the family level, Table S1), *Flavobacteria* and *Cyanobacteria* sequences (Fig. 2a) dominated pelagic communities. In contrast, *Gammaproteobacteria* (25±14.6%) and *Deltaproteobacteria* (16±11.8%) dominated benthic communities. The latter also showed several intermediate abundance taxa including *Flavobacteria, Actinobacteria* and *Betaproteobacteria* that also occurred in pelagic samples, however, at smaller proportions. Other benthic populations such as *Acidobacteria, Planctomycetacia, Clostridia* and *Bacilli* were absent or present in only very low numbers in pelagic samples (Fig. 2a). This was confirmed by evenness estimates at this taxonomic resolution (Simpson's Inverse Index of Diversity  = 3.1±0.82 and 5.4±1.75 for pelagic and benthic samples, respectively; Mann-Whitney rank sum test *P*<0.001).

We further characterised bacterial community composition in each ecosystem type (Fig. 2b), including vent and anoxic ecosystems. Cyanobacterial sequences were noticeably more abundant in open ocean surface waters (13±11.1%) than in coastal waters (3±7.4%). In contrast, deep-water samples showed higher relative sequence abundances of *Deltaproteobacteria* and *Actinobacteria* (Fig. 2b) compared to the surface. Interestingly, the proportion of *SAR11* sequences was similar in surface and deep open ocean ecosystems (47±19.4% and 47±18.6% of the sequences identified at the family level, respectively; Table S1). *SAR11* was, however, less frequently found in coastal samples (29.2±19.4% of the sequences identified at the family level; Table S1), while flavobacterial sequences were particularly abundant (17±12.5%). Alphaproteobacterial sequence abundance was reduced in benthic and anoxic ecosystems, where gammaproteobacterial sequences dominated instead (Fig. 2b). Vent waters and sediments showed clear differences to all other ecosystems in having higher proportions of epsilonbacterial sequences. Anoxic waters also differed, especially from other pelagic ecosystems by having a higher proportion of deltaproteobacterial sequences, as may be expected with sulfate reducers (e.g. *Desulfovibrionales* accounted for 18±1.3% of the total sequences identified at the order level, Table S1). Coastal and deep-sea sediments were only distinguishable by a higher proportion of *Clostridia* and *Bacilli* in coastal sediments, and a higher proportion of *Acidobacteria*, *Planctomycetacia* in deep-sea sediments.

### Bacterial community composition and structure among realms and ecosystem types

**Figure 3:**
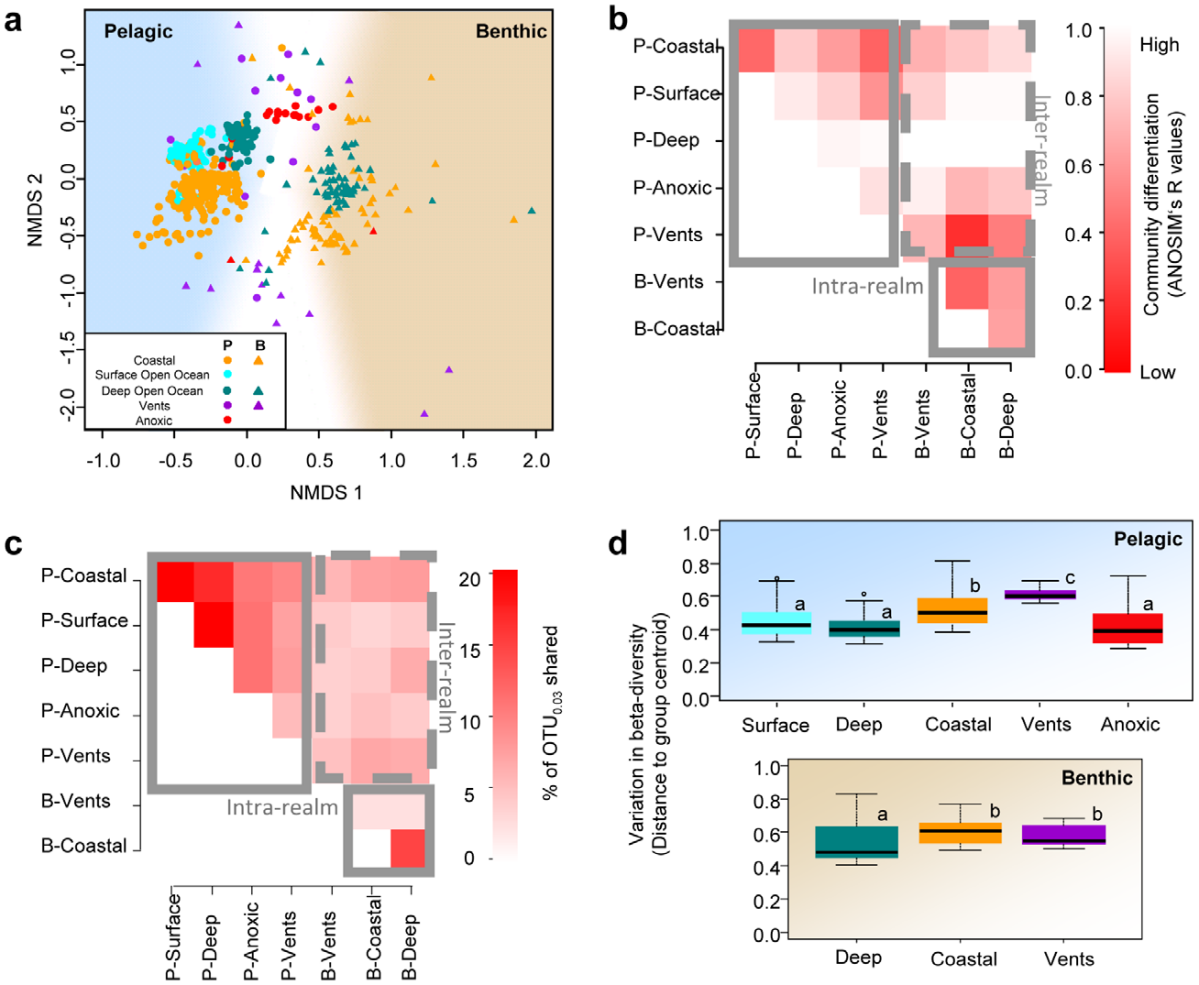
Global beta-diversity patterns of marine bacterial communities according to realms and ecosystem types. (**a**) NMDS ordination of the dissimilarity in bacterial community composition (OTU_0.03_ level, NMDS stress = 0.18). (**b**) Heat map showing pairwise community overlap (upper-half symmetric matrix displayed) according to realm/ecosystem. All ANOSIM R values were significant (1000 Monte Carlo permutations, Bonferroni-corrected *P<*0.05). (**c**) Heat map of the average number of OTU shared between ecosystems types, based on random resampling of 100 subdatasets. (**d**) Boxplots of the variation in beta-diversity in each ecosystem type. Different letters on each box represent significant differences in variance homogeneity between ecosystem types as determined by Mann–Whitney rank sum tests followed by Bonferroni correction (*P<*0.01).

The resulting NMDS ordination highlighted marked bacterial community differences between the pelagic and benthic realms even by removing rare taxa or by using presence/absence data (Fig. 3a, see Material and Methods). This was further confirmed by an Analysis of Similarity of pelagic and benthic communities (ANOSIM, R = 0.56, *P<*0.001, see Material and Methods for further tests on the effect of group size and cell amount). Pairwise ANOSIMs were also used to look at the degree of separation between bacterial communities associated with each ecosystem type (Fig. 3b). The inter-realm community comparisons between ecosystems confirmed the strong differences among pelagic and benthic communities (all ANOSIM's R>0.7; Bonferroni-corrected *P*<0.05 for these comparisons). Inter-realm differences were, however, reduced when comparing vent water to benthic communities (Fig. 3b). On average, higher values of ANOSIM's R were observed for intra-realm comparisons, especially in the pelagic realm (averaged ANOSIM's R ≈ 0.73 and 0.54 for intra-pelagic and intra-benthic comparisons, respectively; all Bonferroni-corrected *P* values <0.05).

### Number of OTUs shared between realms and ecosystem types

To obtain deeper insights into the differences in bacterial community composition, we also assessed the number of shared OTUs between the realms and the ecosystems surveyed (Fig. 3c). By using resampling approaches (see Material and Methods), pelagic and benthic bacterial communities were found to share on average only 7.1±0.01% of their OTU_0.03_ (i.e. ∼9,900 OTU_0.03_). Inter-realm comparisons showed that coastal water associated communities shared on average 7.2±0.02% OTU_0.03_ (i.e. ∼5,600 OTU_0.03_) with benthic communities. Only 4.3±0.02% OTU_0.03_ (i.e. ∼1,800 OTU_0.03_) were shared between pelagic and benthic communities in the other ecosystems (Fig. 3c, Table S2). In parallel, intra-realm comparisons revealed a higher percentage of shared OTU_0.03_ between the different ecosystems when excluding vent and anoxic ecosystems (17.8±0.01% on average). Among the latter ecosystems, vents and in particular their seafloor communities shared the lowest number of OTU_0.03_ (Fig. 3c) with other ecosystems.

### Variability of bacterial community composition in each ecosystem

The variability of bacterial community composition within an ecosystem can provide insights into the strength of environmental filtering or of habitat heterogeneity. Here, we assessed the homogeneity of bacterial community composition within each ecosystem type (Fig. 3d). Overall, the composition and abundance of OTU_0.03_ tended to be more variable for benthic bacterial communities than for pelagic communities. Within each realm, vent and coastal ecosystems systematically displayed the most heterogeneous bacterial communities.

### Relationships with ecosystem type, geographical location and upper water productivity

**Figure 4:**
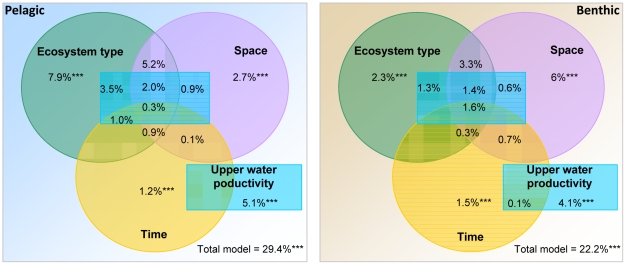
Global beta-diversity patterns of marine bacterial communities within each realm. The respective contributions of ecosystem type, geographic location (transformed latitudes and longitudes, water depth), time (number of days since the first sampling) and upper water productivity (as defined by Longhurst's primary production index and classes of capture fisheries yield) were assessed by using variation partitioning and are displayed as a Venn diagram. The blue boxes (i.e. upper water productivity) correspond to the fourth category of the model, which cannot be represented by a circle in such a display. The significance of each pure effect was validated by performing partial RDA with 1000 Monte Carlo permutation tests. Significance levels: *** *P*<0.001, **P<0.01, *P<0.05. Covariation parts cannot be tested for significance because they are numerically deduced from the pure parts [74].

We disentangled the effects of ecosystem type, spatial location, sampling date, and ecosystem productivity on bacterial community variation in pelagic and benthic realms (Fig. 4). Because the ICoMM dataset includes samples from individual projects that were collected at different dates, the corresponding environmental parameters (e.g. salinity, temperature, NH_4_^+^ concentrations), when provided, represent independent measurements, often based on different methods, limiting global analyses of the entire dataset. Therefore, proxies for long-term regional characterisation were used as described below to reflect ecosystem type and upper water productivity. Time (i.e. number of days since earliest sampling) was also included in the analysis to determine the effects of having different dates of sample collection, which cannot be avoided due to the large spatial extent of the study. Spatial locations were defined using converted latitudes and longitudes into metric distances, as well as water depth. To categorize long-term regional variation in upper water productivity, we used Longhurst productivity indices within Longhurst's provinces and also indices of capture fisheries yields within fisheries areas.

The pure effects of environmental variables (i.e. the amounts of variation explained by these variables while keeping other variables constant) on bacterial community variation were highly significant (Fig. 4; *P*<0.001 with 1000 Monte Carlo permutations), even if coarse environmental descriptors were used in the study. The full models explained 29.4 and 22.2% of the changes in pelagic and benthic communities, respectively (*P*<0.001). Ecosystem type accounted for 7.9 and 2.3% of the total community variation for the pelagic and benthic realms, while spatial variables (distance-converted latitudes and longitudes and water depth) accounted for 2.7 and 6.0%, respectively (Fig. 4). Interestingly, we found a systematic decrease of the spatial effects when only water depth was included in the model (Table S3).

Upper water productivity (Longhurst indices) explained 5.1% of the global pelagic community variation and 4.1% in the benthic realm. When replacing productivity variables in the original model (Table S3) by capture fisheries yields, the latter equally explained the variation in pelagic and benthic communities (about 3% of the explained variation). In contrast, Longhurst's productivity indices better explained the variation in benthic community composition (Table S3). Finally, the differences in sampling date across the ICoMM projects explained only a small fraction of the total variation in bacterial community composition (about 1.5% of the explained variation) in both realms, and hence was not further regarded.

### Consistency of bacterial community distribution at all taxonomic resolution levels

**Figure 5:**
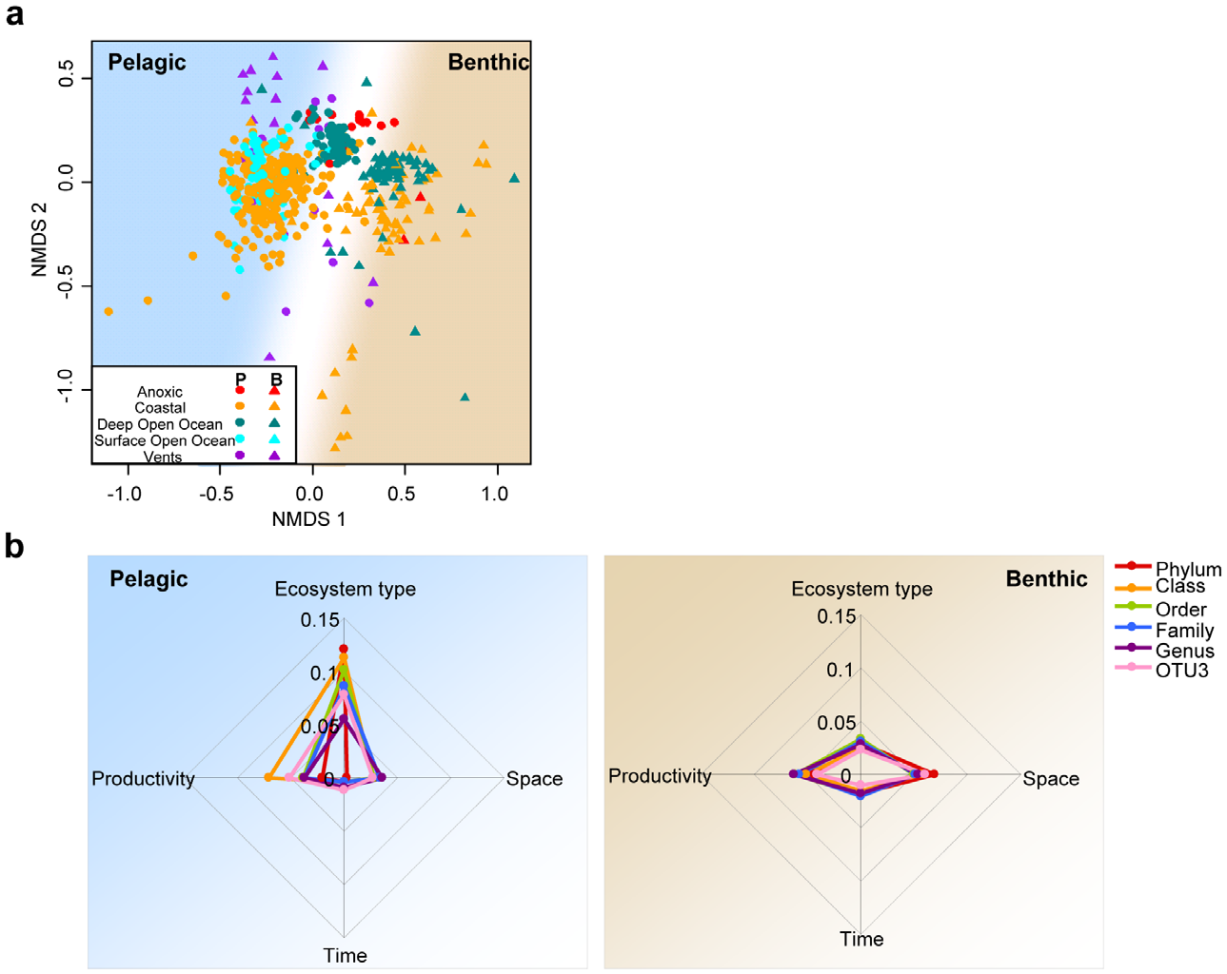
Consistency of patterns of community variation at various taxonomic levels. (**a**) NMDS ordination of the dissimilarity in bacterial community composition at the Phylum level (NMDS stress  = 0.15). The goodness-of-fit between this ordination and the ordination presented in Fig. 3a is shown in Table S4. Pelagic and benthic origins are indicated by the letters P and B, respectively. (**b**) Partitioning of the variation of bacterial composition at different taxonomic levels using the same approach as presented in Fig. 4. Units are expressed in adjusted R^2^ values, i.e. in proportion of explained variance.

We used Procrustes analyses to determine the degree of concordance between NMDS ordinations obtained at different taxonomic resolution levels. Bacterial beta-diversity patterns were strongly reproducible at all taxonomic levels (Table S4, Figs. 3a, 5a), despite decreasing number of annotated OTUs with increasing taxonomic depth (Table S1). Accordingly, taxonomic level had minimal impact on the amount of explained community variation by environmental parameters (Fig. 5b). The trends remained similar, varying slightly in the variance explained (i.e. adjusted R^2^ values), especially for pelagic communities. For the global analysis, the proportion of explained variance was similar across taxonomic levels in the pelagic realm, ranging from 29.4 to 31.6% (genus excluded), and was the highest at the OTU_0.03_ (22.2%) levels for the benthic realm (Fig. 5b).

## Discussion

This synthesis of the ICoMM bacterial dataset provides the most comprehensive picture of global ocean bacterial community distribution to date, including a sampling depth range of 0–5500 m and both pelagic as well as benthic ecosystems. From 509 global samples (Fig. 1, Table 1) we obtained a total ∼120,000 OTU_0.03_, corresponding roughly to ∼80,000 OTU_0.03_ when assuming that species richness is overestimated by 30% due to sequencing errors [38]. This is by far larger than the thousand bacterial types observed in the GOS samples (Global Ocean Sampling Expedition, [13], [14]), but also far below the millions of bacterial types predicted by log-normal models [39], suggesting that although our sampling effort has improved, our approach is still far from recovering the full extent of the predicted bacterial diversity (which is of course still an unverified prediction). Globally and across all ecosystems, about half of all OTU_0.03_ were singletons, but they represented a small proportion of all sequences (Table 1). Such a high proportion of singletons is typically within the range known from other diverse taxa such as tropical arthropods [10], [40], for which undersampling biases are acknowledged [41]. The ICoMM dataset therefore displays rarity features that are similar to what is known for complex communities of macroorganisms, consistent with our conclusion that most of the rare taxa were real rather than sequencing artifacts. Overall, benthic communities seemed more diverse (Table 1) and were more even than pelagic communities, as previously suggested [42]. This may result from the substantially higher density of bacterial populations in sediments, greater habitat temporal stability, higher niche diversity and resource partitioning in the benthic realm, characteristics generally acknowledged to promote both species diversification and coexistence [43].

In the marine bacterial communities investigated here, a large proportion of pelagic and benthic ribosomal sequences belonged to the *Gammaproteobacteria*, supporting observations of their global distribution [20], [22]. This phylum displays a large phylogenetic and phenotypic diversity [44] that may explain the colonization of a large range of ecological niches. The proportion of sequences represented by this clade was lower in the pelagic compared to the benthic realm. Ocean waters were dominated by alphaproteobacterial sequences, comprising primarily free-living and oligotrophic *SAR11* cluster representatives. Furthermore, a high proportion of *Flavobacteria* and *Cyanobacteria* were detected (Fig. 2). These proportions are consistent with earlier reports [1], [45], and attest of the quality and ecological relevance of the data presented here.

For benthic communities this study provides the first overview of their global composition. The dominant groups were *Gammaproteobacteria*, *Deltaproteobacteria*, *Planctomycetes*, *Actinobacteria*, and *Acidobacteria* (Fig. 2). These clades comprise many microaerophilic or anaerobic heterotrophs and chemoautotrophs, with the *Deltaproteobacteria* apparently predominantly represented by sulfate reducers. Our study thus confirms and extends what has been reported in terms of composition of benthic bacterial communities from subseafloor or coastal sediments at more local or regional scales [46], [47], and supports the idea that the composition of pelagic and benthic communities differ [26], at the global scale.

The differences between pelagic and benthic realms also held at the much finer level of community resolution, i.e. that of OTU_0.03_ level (Fig. 3), as previously suggested based on smaller datasets [42]. This result was confirmed either when removing all singletons, or when simulating a wide range of differences in sample numbers and sequence proportions between realms. Moreover, pelagic and benthic bacterial communities shared only a small fraction of their OTU_0.03_ (i.e. ∼9,900 OTU_0.03;_ <10% of all bacterial types). Finding such differences between pelagic and benthic bacterial communities may sound intuitive at first sight. However, the low overlap between pelagic and benthic communities detected in this global scale study is surprising, given the origin of marine surface sediments from sinking inorganic and organic particles which transport and deposit surface-borne bacteria at depth [24].

Differences between pelagic and benthic communities were also observed when ecosystem types were compared within each realm, but inter-realm differences dominated over intra-realm community differences (Fig. 3a–c). Noticeably, bacterial communities originating from hydrothermal vent and anoxic waters displayed both pelagic and benthic characteristics (Figs. 2b, 3a–b). In vent ecosystems, we detected high proportions of *Gammaproteobacteria* and *Epsilonproteobacteria* (Fig. 2b), both hosting characteristic members of these habitats, such as the sulphur oxidizers [42]. Both taxa are known to be highly versatile both morphologically and phylogenetically [44], [48]. Accordingly, vent communities shared the lowest fraction of their OTU_0.03_ with other ecosystems, pelagic or benthic vent included (Fig. 3c). Therefore they displayed the highest variability (Fig. 3d), especially when considering that vent samples originated from only few geographic locations in this study (Fig. 1). This variability in bacterial community composition is in line with the high variability of fluid emission, distribution and interactions with the bio- and geosphere occurring in hydrothermal vent ecosystems [49], and might be increased by their “island” nature. Indeed, as vent ecosystems are highly fragmented habitats and very patchily distributed at the global scale, one may expect higher adaptation of their communities to the local conditions. Anoxic ecosystems displayed higher proportions of *Deltaproteobacteria*, comprising many anaerobes such as the sulfate-reducing bacteria, and a large dominance of *Gammaproteobacteria* (Fig. 2), which is also in accordance with earlier reports [50].

Vents and anoxic water samples displayed communities similar to both sediments and water samples (Fig. 3a), which partly reflect large difference in redox state of these microbial habitats [42], [50]. Oxygen depletion and the availability of highly reduced chemical compounds could favour bacterial populations with genetic or phenotypic adaptations to life at chemoclines and in anoxia [42], [50], conditions that are characteristic of most benthic ecosystems. Furthermore, this result may also reflect the contrasting conditions between the seafloor and seawater, e.g. surfaces availability for colonization and organism lifestyles [42], organism density as well as predation mechanisms.

In open ocean waters, the observed vertical distribution patterns (Fig. 2b, 3a) were very similar to what has been described so far [1], [11], [51], namely (i) the decrease of *Cyanobacteria* with depth due to their dependence on light availability; (ii) the increase of *Deltaproteobacteria* and *Actinobacteria*; and (iii) the abundance of the *SAR11* cluster across all depths, whose members display vertical stratification [12]. Open ocean surface and deep waters shared the highest proportion of OTU_0.03_ (Fig. 3c), and showed reduced community variability compared to benthic communities, regardless of geographic location (Fig. 3d), suggesting stronger mixing and environmental filtering.

Coastal water communities appeared more related to open ocean surface water communities (Fig. 3a–c), but displayed higher proportions of *Flavobacteria* and lower proportions of *SAR11* and *Cyanobacteria* (Fig. 2b). This observation contrasts with an earlier study based on the GOS data, which had focused on pre-filtered free-living organisms in mostly warm surface waters [21]. *Flavobacteria* are indeed enriched on detritus particles in coastal waters [45], [52], which may partly explain why they are relatively abundant in unfiltered water samples. Furthermore, the observed decrease of *Cyanobacteria* and *SAR11* sequence relative abundance in coastal habitats corresponds with local higher nutrient availability and dominance of eukaryotic phytoplankton, which is assumed to exert strong competitive pressure on phototrophic bacterioplankton [13], [53]. Finally, coastal waters shared higher proportion of OTUs_0.03_ with benthic communities (Fig. 3c) and may harbour benthic taxa resuspended in the rather shallow water column through upwelling currents and storms. Alternatively, some of these differences might also result from the influence of land, providing higher freshwater, nutrient and organic matter input to coastal waters [54] and sediments.

Bacterial communities associated with coastal sediments were highly variable (Fig. 3d). Interestingly, *Firmicutes* (*Bacilli* and *Clostridia*) occurred in higher proportions in coastal sediments. These two clades have been recently identified as indicators of human faecal contaminations in watersheds [55], but are also common taxa in soils [56]. Since they are almost absent in the other marine ecosystem types, one may hypothesize that they have a terrestrial origin and might not be adapted to aquatic lifestyle or to the presence of dissolved oxygen in the water column, as many members of these clades are anaerobes. In general, coastal habitats have temporally and spatially variable physico-chemical factors, which may explain the high variability of bacterial communities observed in both coastal waters and coastal sediments (Fig. 3d). This heterogeneity might increase ecosystem resilience since certain habitat patches may serve as refugia, as previously described for freshwater metazoa [57]. Given the fact that coastal ecosystems are subjected to increasing pollution and habitat loss [58], future research will need to determine how such deleterious effects may impact the functioning of bacterial communities and the processes that they govern.

Deep-sea sediments displayed similar bacterial communities to coastal sediments, although less variable (Fig. 3d), which might reflect the lower environmental dynamics occurring in the generally nutrient-poor deep sea [7]. The most abundant taxa observed here (Fig. 2b) are very consistent with what has been reported in the literature [2], [46]. However, *Chloroflexi* and above all the *JS1* candidate division, usually described as among the most abundant bacterial taxa in the seabed, were only abundant in a few deep-sea sediment samples of this study. These disparities may arise from differences in the depth of seafloor sampling, here mostly confined to the top 10 cm surface sediments. Other studies of deep-sea surface sediments [59], [60], also found more acidobacterial sequences and less of *JS1* and *Chloroflexi*, suggesting that these taxa could be indicators of differences between surface and subsurface benthic realms.

We further used a variation partitioning approach to identify the contribution of different factors in the distribution of bacterial communities. A previous synthesis of global bacterial biomass data showed that a combination of broad proxies for ocean realms and their productivity had a very high predictive force, in contrast to water depth alone [61]. Due to the extent and complexity of the individual projects comprised in ICoMM, we also used combinations of proxy variables [61] in this synthesis of global bacterial community patterns (see Results and Material and Methods sections), namely: (i) Ecosystem type, to represent the degree of habitat determinism, (ii) spatial components, to represent the degree of provincialism, and (iii) ocean productivity, which is represented by both phytoplankton productivity and capture fisheries yield indices so as to integrate regional resources at the two extremes of the food chain and to provide a link to ecosystem services.

Ecosystem type and geographic distance had contrasting effects on pelagic and benthic community composition (Fig. 4, Table S3). While we could not completely test the Baas-Becking and Biejerinck hypothesis, we noted that identical, yet remote habitats harbour similar communities in the pelagic realms. In contrast, spatial distances were found to considerably explain changes in benthic community composition, probably because horizontal physical mixing is much more limited at the seafloor than in seawater. As a result, many environmental variables may be more spatially autocorrelated and microbial communities may also be more subject to spatial isolation. When replacing the spatial descriptors by water depth only, the latter appeared to have a minor influence on both pelagic and benthic bacterial community distributions (Table S3), as previously observed for the distribution of bacterial biomass in sediments [61]. Furthermore, our study suggests that previous contradicting hypotheses about microbial cosmopolitanism [28] or endemism [22] may have resulted from relatively limited sampling effort across ecosystem types. Therefore, contrasted biogeographic patterns may not be contradictory if one considers a continuum from cosmopolitanism to endemism with many intermediate distribution patterns as a function of habitat type and heterogeneity. This is consistent with what has been described for a large range of macroorganisms [23], and clearly shows that bacterial communities may also display remarkable large-scale, beta-diversity patterns.

The variations in capture fishery yields produced similar levels of explained variation for both pelagic and benthic bacterial communities (Table S3, Fig. 4). This suggests that not only seafloor bacterial biomass, but also its community composition depend on fluxes from the euphotic zone [6], [62]. For pelagic communities, the weak relationship detected between community patterns and Longhurst's productivity indices might be due to the highly dynamic coupling between productivity and bacterial community composition that is known to exist on small temporal and spatial scales [63]. Using world ocean atlas data and remote sensing estimates of productivity at different spatial or temporal scales [61] could be important next steps to help reveal specific relationships between bacterial diversity and different water masses, productivity, or other environmental characteristics.

Finally, the observed beta-diversity patterns and their ecological interpretation were supported at all taxonomic levels investigated (Fig. 5, Table S4). Contrarily to what is often thought, identification at finer taxonomic ranks may not be always necessary to provide a meaningful understanding of biological variation or ecological phenomena. This may be due in part to the noise generated by numerous minor groups detected at finer taxonomic resolution levels, especially for microbes and their characteristic long-tailed rank-abundance distributions [64]. Overall, our results strengthen the idea that observations made on broad taxonomic ranks are ecologically meaningful [33] for marine microbial communities and suggest that the patterns observed at the global scale may result from long-term processes. Further, our results imply that the minority of taxonomically annotated sequences, which constituted only 24% of the total dataset at the Genus level (Table S1), still carries important ecological signal (Fig. 5). Although future investigations are needed to disentangle the ecological and evolutionary processes underlying these patterns, the distinct community composition and distribution observed in the pelagic and benthic realms and across all taxonomic ranks, as well as the significant beta-diversity patterns revealed here, demonstrate that it is possible to define broad biomes for marine microbes.

## Materials and Methods

### Dataset description

The ICoMM 454 bacterial 16S pyrotag dataset and geospatial parameters, namely latitudes, longitudes and water depth are available on the web (VAMPS website: http://vamps.mbl.edu, MICROBIS website: http://icomm.mbl.edu/microbis) and are provided in Table S5. Based on sample location, water depth and initial description, we defined five ecosystem types classically used in oceanography, namely anoxic and vents on the basis of sample description provided by the MICROBIS website, coastal (<200 nautical miles [nmi] from the littoral), deep seafloor (samples >200 m water depth), deep waters (>200 nmi from the coast and >200 m water depth) and surface waters (>200 nmi from the coast and <200 m water depth). We retrieved primary production rate data and their corresponding primary productivity indices (http://www.vliz.be/vmdcdata/vlimar/downloads.php) from the Longhurst biogeographical provinces [65]. Capture fishing data were collected from the FAO Fisheries and Aquaculture Statistics and Information (www.fao.org/fishery/statistics/software/fishstat/en), log-transformed and converted into classes. We assigned these indices to samples according to the FAO fishing area (http://www.vliz.be/vmdcdata/vlimar/downloads.php).

### Generation and taxonomic annotation of pyrotags

DNA extraction procedures are reported on the project pages of the MICROBIS website. For all DNA extracts, the hypervariable V6 region of the bacterial 16S rRNA gene was amplified using a set of five forward and four reverse PCR primers (http://vamps.mbl.edu/resources/faq.php#tags). PCR products were submitted to massively parallel tag sequencing using a 454 Life Sciences GS FLX sequencer at the Marine Biological Laboratory in Woods Hole, Massachusetts. Low quality sequences, i.e. sequences <50 nt length, containing ambiguous nucleotides, non-exact key/forward primers were removed [36]. The sequences were deposited in GenBank Sequence Read Archives (www.ncbi.nlm.nih.gov *)* and their accession numbers are provided in Table S5. Each sequence was taxonomically assigned using an updated version of the GAST pipeline, previously developed by [66]. All sequences obtained from the 509 samples (Table 1) were clustered into OTU_0.03_ as described previously [37] and all sequences that did not belong to the domain *Bacteria* were discarded from the analysis. As far as clustering and noise removal in the pyrotag sequences are concerned, OTU clustering results using pairwise alignments and the single-linkage preclustering followed by average linkage clustering are equivalent to those using the PyroNoise software, as previously demonstrated by both Quince et al. [38] (see Figure 3 in that reference) and Huse et al [37]. Nevertheless, although useful to reduce the amount of technical noise, both methods cannot completely correct for sequencing or PCR errors [38].

### Diversity analysis

Taxa/OTU-abundance tables obtained from the pyrotag taxonomic assignment or sequence clustering were standardised by Hellinger transformation and dissimilarities between all pairs of samples were calculated using Bray-Curtis dissimilarity coefficient in order to obtain a beta-diversity matrix [67]. The resulting distance matrix was reduced in a 2D-space by using non-metric multidimensional scaling (NMDS) with 20 random starts. To ensure that the presence of rare sequences did not affect ecological interpretations we evaluated the effects of the high number of singletons (Table 1) on bacterial community distribution at the OTU_0.03_ level by we analysing the datasets with or without singletons as follows: Bray-Curtis distances between samples were calculated, 2-dimensional NMDS ordinations were generated, and pairwise comparisons of the ordination solutions were done using Procrustes correlation analysis [68]. Bacterial community distribution was not affected by the high number of singletons (Procrustes correlation coefficient = 0.997, *P*<0.001, indicating nearly similar ordination results). Similarly when we tested the effects of using presence/absence vs. relative abundance data, the resulting NMDS ordinations were highly correlated with one another (Procrustes correlation coefficient  = 0.909, *P*<0.001). Hence, subsequent analyses were performed using relative abundance data and Bray-Curtis distances, including singletons in the calculations.

Analyses of similarities (ANOSIM, [67]) were performed to test for significant differences between groups of samples using 1000 Monte Carlo permutation tests. Because ANOSIM may be sensitive to differences in group size, we conducted simulations of equal sample size per realm (n = 140) by resampling the original dataset 1,000 times and repeating the ANOSIM on these subsets with 100 Monte Carlo permutation tests. Due to computing limitations, this analysis was restricted to the Class level only, although we expect the OTU_0.03_ level to yield similar results (see below). These simulations also yielded highly significant R values that ranged from 0.557 to 0.620. Similarly, we tested the effects of attributing varying weights to water and sediment samples so as to mimic the effects of differences in individual cell densities between realms [3] on our ecological interpretations. Indeed, it is inherently difficult to obtain a standardized comparison of sediment and water samples, as it is generally left to the discretion of the investigators to choose the best amount or volume of starting material for molecular analyses. We thus allowed sequence abundance data to vary over three orders of magnitude and tested the degree of separation in the resulting communities. All ANOSIM's R values were highly significant (P<0.001) and ranged from 0.907 to 0.912, thus indicating that even higher levels of community separation between the pelagic and benthic realms were obtained when simulating more heterogeneity in the experimental procedures.

The extent of community turnover was determined by calculating the average variation in bacterial beta-diversity for each sample group to its centroid [69], which was then compared between groups using Mann-Whitney rank sum tests. The consistency of bacterial beta-diversity patterns at all taxonomic ranks was statistically verified by pairwise comparisons of NMDS ordinations using Procrustes analyses, and assessed for randomness by 1000 Monte Carlo permutation tests followed by Bonferroni correction for multiple testing.

RDA (Redundancy Analyses, for the Phylum, Class, Order, Family and Genus levels, [67]) and distance-based RDA (db-RDA [67], for the OTU_0.03_ level) were carried out to evaluate the combined effects of ecosystem type, space, and sampling date on bacterial community composition in both realms. To this end, the linear distance matrix of converted longitude and latitude coordinate vectors that took the earth curvature into account was converted to an Euclidean plane by using a rigid rotation of the axes (i.e. by principal coordinate analysis), so as to select for main axes that represented the largest directions of spatial variation. Variation partitioning was carried out to estimate the pure effects of each explanatory factor on bacterial beta-diversity [70]. The significance of each pure effect was assessed by performing partial RDA with 1000 Monte Carlo permutation tests. All analyses were carried out with the R statistical environment [71], with the packages *vegan* [72], and *gmt* [73], as well as with custom R scripts.

## Supporting Information

Table S1 **Sequence characteristics at each taxonomic level.**
(DOC)

Table S2 **Percentage of shared OTU_0.03_ between the different ecosystem types.**
(DOC)

Table S3 **Variation of bacterial composition (OTU_0.03_) explained by various contextual parameters.**
(DOC)

Table S4 **Consistency of beta-diversity patterns across taxonomic levels.**
(DOC)

Table S5 **Datasets, location and parameters used in this study.**
(DOC)
