# Supplementary material for: Global Patterns of Bacterial Beta-Diversity in Seafloor and Seawater Ecosystems
Source: PLoS One. 2011 Sep 8;6(9):e24570. doi: 10.1371/journal.pone.0024570 (PMC3169623; doi:10.1371/journal.pone.0024570)
Supplement: Table S1 — Sequence characteristics at each taxonomic level. (DOC) [file pone.0024570.s001.doc]

**Table S1. Sequence characteristics at each taxonomic level.**

|  | Number of sequences | Number of taxa | Mean assigned (%) |
| --- | --- | --- | --- |
| Phylum | 9,108,575 | 41 | 95.0 |
| Class | 8,168,665 | 40 | 85.2 |
| Order | 5,539,818 | 97 | 57.8 |
| Family | 4,771,150 | 245 | 49.8 |
| Genus | 2,323,492 | 953 | 24.2 |
| OTU_0.10_* | 9,587,850 | 64,416 | n.a. |
| OTU_0.06_* | 9,587,850 | 89,665 | n.a. |
| OTU_0.03_* | 9,587,850 | 120,436 | n.a. |

*OTU_0.03_, OTU_0.06_ and OTU_0.10_ represent the OTU obtained at 3, 6 and 10% of sequence dissimilarity threshold.

n.a.: not applicable. Number of samples = 509
