# Supplementary material for: Global Patterns of Bacterial Beta-Diversity in Seafloor and Seawater Ecosystems
Source: PLoS One. 2011 Sep 8;6(9):e24570. doi: 10.1371/journal.pone.0024570 (PMC3169623; doi:10.1371/journal.pone.0024570)
Supplement: Table S2 — Percentage of shared OTU0.03 between the different ecosystem types. (DOC) [file pone.0024570.s002.doc]

**Table S2. Percentage of shared OTU_0.03_ between the different ecosystem types**

| Ecosystem type | P-Surface | P-Deep | P-Anoxic | P-Vents | B-Vents | B-Coastal | B-Deep |
| --- | --- | --- | --- | --- | --- | --- | --- |
| P-Coastal | 18.9 ± 0.21 | 15.7 ± 0.12 | 10.1 ± 0.22 | 8.7 ± 0.35 | 5.4 ± 0.09 | 7.1 ± 0.02 | 7.4 ± 0.02 |
| P-Surface |  | 18.8 ± 0.11 | 10.5 ± 0.20 | 6.9 ± 0.37 | 3.9 ± 0.09 | 3.2 ± 0.01 | 3.8 ± 0.02 |
| P-Deep |  |  | 10.2 ± 0.24 | 7.4 ± 0.17 | 3.5 ± 0.07 | 4.1 ± 0.01 | 6.2 ± 0.02 |
| P-Anoxic |  |  |  | 5.2 ± 0.08 | 3.4 ± 0.08 | 4.6 ± 0.03 | 3.9 ± 0.03 |
| P-Vents |  |  |  |  | 4.6 ± 0.19 | 6.7 ± 0.01 | 6.1 ± 0.08 |
| B-Vents |  |  |  |  |  | 2.6 ± 0.03 | 2.6 ± 0.05 |
| B-Coastal |  |  |  |  |  |  | 13.7 ± 0.03 |

Values represent the average percentage of shared OTU_0.03_ ± standard deviation.

P and B refer to Pelagic and Benthic, respectively.
