# Supplementary material for: Global Patterns of Bacterial Beta-Diversity in Seafloor and Seawater Ecosystems
Source: PLoS One. 2011 Sep 8;6(9):e24570. doi: 10.1371/journal.pone.0024570 (PMC3169623; doi:10.1371/journal.pone.0024570)
Supplement: Table S3 — Variation of bacterial composition (OTU0.03) explained by various contextual parameters. (DOC) [file pone.0024570.s003.doc]

**Table S3. Variation of bacterial composition in OTU_0.03_ explained by various contextual parameters**

| Models | Total explained  (Y) | Ecosystem type  (E) | Spatial location  (S) | Time  (T) | Productivity  (P) | Covariation  (C) |
| --- | --- | --- | --- | --- | --- | --- |
| Pelagic |  |  |  |  |  |  |
| Y=E+S+T+P1+C | 25.8% | 7.7% | 3.5% | 0.9% | 1.4% | 12.4% |
| Y=E+S+T+P2+C | 27.9% | 11.0% | 2.6% | 1.3% | 3.5% | 9.4% |
| Y=E+S1+T+P+C | 28.9% | 12.1% | 2.1% | 1.2% | 5.1% | 8.4% |
| Y=E+S2+T+P+C | 27.3% | 8.8% | 0.6% | 1.2% | 6.0% | 10.7% |
| Benthic |  |  |  |  |  |  |
| Y=E+S+T+P1+C | 22.2% | 2.3% | 6.0% | 1.1% | 4.2% | 8.6% |
| Y=E+S+T+P2+C | 21.1% | 3.8% | 7.0% | 1.3% | 3.0% | 5.9% |
| Y=E+S1+T+P+C | 19.6% | 2.5% | 6.0% | 1.2% | 1.5% | 8.3% |
| Y=E+S2+T+P+C | 18.4% | 1.9% | 2.2% | 1.5% | 5.0% | 7.7% |

The original model is presented in Fig. S2. E = ecosystem type, S = PCoA-transformed latitudes and longitudes, water depth, T = number of days since the oldest sampling, and P = Longhurst’s primary production indices and capture fisheries yields. Different combinations of spatial variables or productivity were replaced in the original model: S1= PCoA-transformed latitudes and longitudes; S2 = water depth, P1 = Longhurst’s productivity indices, P2 = capture fisheries yields. R^2^ values are expressed here as percentage of explained variance. Significance of the pure effect of each parameter was assessed by 1000 Monte Carlo permutation tests. All adjusted R^2^ values were found highly significant (*P*<0.001). Covariation are not testable (See Fig. 4)
