# Supplementary material for: Global Patterns of Bacterial Beta-Diversity in Seafloor and Seawater Ecosystems
Source: PLoS One. 2011 Sep 8;6(9):e24570. doi: 10.1371/journal.pone.0024570 (PMC3169623; doi:10.1371/journal.pone.0024570)
Supplement: Table S4 — Consistency of beta-diversity patterns across taxonomic levels. (DOC) [file pone.0024570.s004.doc]

**Table S4. Consistency of beta-diversity patterns across taxonomic levels**

|  | Class | Order | Family | Genus | OTU_0.10_ | OTU_0.06_ | OTU_0.03_ |
| --- | --- | --- | --- | --- | --- | --- | --- |
| Phylum | 0.67 | 0.69 | 0.71 | 0.65 | 0.67 | 0.69 | 0.69 |
| Class |  | 0.89 | 0.85 | 0.76 | 0.74 | 0.75 | 0.75 |
| Order |  |  | 0.93 | 0.80 | 0.80 | 0.80 | 0.76 |
| Family |  |  |  | 0.85 | 0.84 | 0.85 | 0.82 |
| Genus |  |  |  |  | 0.82 | 0.80 | 0.81 |
| OTU_0.10_ |  |  |  |  |  | 0.99 | 0.98 |
| OTU_0.06_ |  |  |  |  |  |  | 0.98 |

Correlations between NMDS ordinations at different taxonomic levels were assessed by using procrustean analyses. Procrustean correlations were tested for significances by performing 1000 Monte Carlo permutation tests, followed by Bonferroni correction for multiple testing. All valued were significant (*P*<0.05)
