# Supplementary material for: Global Patterns of Bacterial Beta-Diversity in Seafloor and Seawater Ecosystems
Source: PLoS One. 2011 Sep 8;6(9):e24570. doi: 10.1371/journal.pone.0024570 (PMC3169623; doi:10.1371/journal.pone.0024570)
Supplement: Table S5 — Datasets, location and parameters used in this study. (DOC) [file pone.0024570.s005.doc]

**Table S5.** Datasets, location and parameters used in this study

| Dataset | Sequence Archive accession # | Longitude (dd) | Latitude (dd) | Depth (m) | Date | Realm | Ecosystem type | Productivity Longhurst's index | Capture fisheries yield index |
| --- | --- | --- | --- | --- | --- | --- | --- | --- | --- |
| ABR_0001_2005_01_02 | SRA009837.1 | -170.00 | -64.50 | 0 | 02/01/2005 | Water | Open Ocean | 2 | 1 |
| ABR_0005_2005_01_07 | SRA009837.1 | -170.00 | -50.00 | 0 | 07/01/2005 | Water | Open Ocean | 1 | 3 |
| ABR_0009_2005_01_30 | SRA009837.1 | -170.00 | -20.00 | 0 | 30/01/2005 | Water | Open Ocean | 1 | 4 |
| ABR_0013_2005_02_26 | SRA009837.1 | -170.00 | 0.08 | 0 | 26/02/2005 | Water | Open Ocean | 1 | 4 |
| ACB_0001_2007_07_13 | SRA009838.1 | -156.06 | 71.45 | 2 | 13/07/2007 | Water | Coastal | 5 | 1 |
| ACB_0002_2007_07_11 | SRA009838.1 | -156.86 | 71.43 | 2 | 11/07/2007 | Water | Coastal | 5 | 1 |
| ACB_0003_2008_01_26 | SRA009838.1 | -156.68 | 71.35 | 2 | 26/01/2008 | Water | Coastal | 5 | 1 |
| ACB_0004_2008_01_30 | SRA009838.1 | -156.68 | 71.35 | 2 | 30/01/2008 | Water | Coastal | 5 | 1 |
| ACB_0005_2002_07_29 | SRA009838.1 | -151.98 | 72.32 | 10 | 29/07/2002 | Water | Coastal | 5 | 1 |
| ACB_0006_2002_08_06 | SRA009838.1 | -159.34 | 72.24 | 10 | 06/08/2002 | Water | Coastal | 5 | 1 |
| ACB_0007_2004_07_30 | SRA009838.1 | -150.89 | 71.54 | 8.4 | 30/07/2004 | Water | Coastal | 5 | 1 |
| ACB_0008_2004_08_21 | SRA009838.1 | -152.02 | 72.42 | 39.7 | 21/08/2004 | Water | Coastal | 5 | 1 |
| ACB_0013_2004_01_17 | SRA009838.1 | -126.30 | 70.04 | 3 | 17/01/2004 | Water | Coastal | 5 | 1 |
| ACB_0015_2004_07_16 | SRA009838.1 | -126.31 | 70.05 | 1.5 | 16/07/2004 | Water | Coastal | 5 | 1 |
| ACB_0016_2008_01_28 | SRA009838.1 | -156.68 | 71.35 | 2 | 28/01/2008 | Water | Coastal | 5 | 1 |
| AGW_0001_2005_06_15 | SRA009839.1 | -53.18 | 5.54 | 1 | 15/06/2005 | Sediment | Coastal | 5 | 4 |
| AGW_0002_2005_06_15 | SRA009839.1 | -53.18 | 5.54 | 1 | 15/06/2005 | Sediment | Coastal | 5 | 4 |
| AGW_0003_2006_06_15 | SRA009839.1 | -52.20 | 5.54 | 52 | 15/06/2006 | Sediment | Coastal | 5 | 4 |
| AGW_0004_2006_06_15 | SRA009839.1 | -52.20 | 5.54 | 52 | 15/06/2006 | Sediment | Coastal | 5 | 4 |
| ALR_0001_2005_04_09 | SRA009840.1 | -176.14 | -20.32 | 2707 | 09/04/2005 | Sediment | Vents | 1 | 4 |
| ALR_0002_2005_04_11 | SRA009840.1 | -176.14 | -20.32 | 2714 | 11/04/2005 | Sediment | Vents | 1 | 4 |
| ALR_0003_2005_04_13 | SRA009840.1 | -176.19 | -20.76 | 2139 | 13/04/2005 | Sediment | Vents | 1 | 4 |
| ALR_0004_2005_04_16 | SRA009840.1 | -176.60 | -22.18 | 1908 | 16/04/2005 | Sediment | Vents | 1 | 4 |
| ALR_0006_2005_04_22 | SRA009840.1 | -176.60 | -22.18 | 1918 | 22/04/2005 | Sediment | Vents | 1 | 4 |
| ALR_0007_2005_04_24 | SRA009840.1 | -176.57 | -21.99 | 1875 | 24/04/2005 | Sediment | Vents | 1 | 4 |
| ALR_0008_2005_05_04 | SRA009840.1 | -176.13 | -20.05 | 2619 | 03/05/2005 | Sediment | Vents | 1 | 4 |
| ALR_0017_2005_04_09 | SRA009840.1 | -176.14 | -20.32 | 2707 | 09/04/2005 | Sediment | Vents | 1 | 4 |
| ALR_0019_2005_04_09 | SRA009840.1 | -176.14 | -20.32 | 2707 | 09/04/2005 | Sediment | Vents | 1 | 4 |
| AOT_0001_2008_11_07 | SRA009841.1 | -11.64 | 42.85 | 47 | 07/11/2008 | Water | Open Ocean | 1 | 5 |
| AOT_0002_2008_11_09 | SRA009841.1 | -13.36 | 37.13 | 1100 | 08/11/2008 | Water | Open Ocean | 1 | 5 |
| AOT_0003_2008_11_09 | SRA009841.1 | -13.36 | 37.13 | 73 | 09/11/2008 | Water | Open Ocean | 1 | 5 |
| AOT_0004_2008_11_14 | SRA009841.1 | -20.50 | 22.51 | 89 | 14/11/2008 | Water | Open Ocean | 1 | 4 |
| AOT_0005_2008_11_16 | SRA009841.1 | -30.00 | 14.76 | 7 | 16/11/2008 | Water | Open Ocean | 1 | 4 |
| AOT_0006_2008_11_29 | SRA009841.1 | 8.52 | -23.71 | 28 | 29/11/2008 | Water | Open Ocean | 3 | 4 |
| AOT_0007_2008_11_17 | SRA009841.1 | -20.36 | 44.70 | 7 | 17/11/2008 | Water | Open Ocean | 2 | 5 |
| AOT_0008_2008_11_17 | SRA009841.1 | -20.13 | 10.63 | 100 | 17/11/2008 | Water | Open Ocean | 1 | 4 |
| AOT_0009_2008_11_17 | SRA009841.1 | -20.13 | 10.63 | 48 | 17/11/2008 | Water | Open Ocean | 1 | 4 |
| AOT_0010_2008_11_17 | SRA009841.1 | -20.06 | 10.37 | 11 | 17/11/2008 | Water | Open Ocean | 1 | 4 |
| AOT_0011_2008_11_17 | SRA009841.1 | -20.06 | 10.37 | 200 | 17/11/2008 | Water | Open Ocean | 1 | 4 |
| AOT_0012_2008_11_17 | SRA009841.1 | -20.06 | 10.37 | 1300 | 17/11/2008 | Water | Open Ocean | 1 | 4 |
| AOT_0013_2008_11_18 | SRA009841.1 | -19.50 | 8.50 | 7 | 18/11/2008 | Water | Open Ocean | 1 | 4 |
| AOT_0014_2008_11_29 | SRA009841.1 | 8.52 | -23.71 | 4600 | 29/11/2008 | Water | Open Ocean | 3 | 4 |
| AOT_0015_2008_11_20 | SRA009841.1 | -14.15 | 2.47 | 45 | 20/11/2008 | Water | Open Ocean | 2 | 4 |
| AOT_0016_2008_11_27 | SRA009841.1 | 3.13 | -17.74 | 48 | 27/11/2008 | Water | Open Ocean | 1 | 4 |
| ASA_0001_2007_12_18 | SRA009843.1 | -115.68 | -73.94 | 9.5 | 18/12/2007 | Water | Coastal | 3 | 1 |
| ASA_0002_2007_12_18 | SRA009843.1 | -115.68 | -73.94 | 9.5 | 18/12/2007 | Water | Coastal | 3 | 1 |
| ASA_0003_2007_12_18 | SRA009843.1 | -115.68 | -73.94 | 19.5 | 18/12/2007 | Water | Coastal | 3 | 1 |
| ASA_0004_2007_12_18 | SRA009843.1 | -115.68 | -73.94 | 19.5 | 18/12/2007 | Water | Coastal | 3 | 1 |
| ASA_0005_2007_12_18 | SRA009843.1 | -115.68 | -73.94 | 34.5 | 18/12/2007 | Water | Coastal | 3 | 1 |
| ASA_0006_2007_12_18 | SRA009843.1 | -115.68 | -73.94 | 34.5 | 18/12/2007 | Water | Coastal | 3 | 1 |
| ASA_0007_2007_12_18 | SRA009843.1 | -115.68 | -73.94 | 49.5 | 18/12/2007 | Water | Coastal | 3 | 1 |
| ASA_0008_2007_12_18 | SRA009843.1 | -115.68 | -73.94 | 99.5 | 18/12/2007 | Water | Coastal | 3 | 1 |
| ASA_0009_2007_12_18 | SRA009843.1 | -115.68 | -73.94 | 99.5 | 18/12/2007 | Water | Coastal | 3 | 1 |
| ASA_0010_2007_12_18 | SRA009843.1 | -115.68 | -73.94 | 249.5 | 18/12/2007 | Water | Coastal | 3 | 1 |
| ASA_0011_2007_12_18 | SRA009843.1 | -115.68 | -73.94 | 249.5 | 18/12/2007 | Water | Coastal | 3 | 1 |
| ASA_0012_2007_12_18 | SRA009843.1 | -115.68 | -73.94 | 495.5 | 18/12/2007 | Water | Coastal | 3 | 1 |
| ASA_0013_2007_12_18 | SRA009843.1 | -115.86 | -73.96 | 784.5 | 18/12/2007 | Water | Coastal | 3 | 1 |
| ASA_0014_2007_12_18 | SRA009843.1 | -115.86 | -73.96 | 499.5 | 18/12/2007 | Water | Coastal | 3 | 1 |
| ASV_0001_1998_05_29 | SRA009844.3 | -25.85 | 37.86 | 0.2 | 29/05/1998 | Water | Vents | 1 | 5 |
| ASV_0002_1998_06_18 | SRA009844.3 | -25.85 | 37.86 | 0.2 | 18/06/1998 | Water | Vents | 1 | 5 |
| ASV_0003_1998_06_25 | SRA009844.3 | -25.85 | 37.86 | 0.1 | 25/06/1998 | Water | Vents | 1 | 5 |
| ASV_0004_1998_06_25 | SRA009844.3 | -25.85 | 37.86 | 0.2 | 25/06/1998 | Water | Vents | 1 | 5 |
| ASV_0005_2008_06_11 | SRA009844.3 | -25.85 | 37.86 | 0.1 | 11/06/2008 | Water | Vents | 1 | 5 |
| ASV_0006_1998_06_24 | SRA009844.3 | -25.31 | 37.73 | 0.8 | 24/06/1998 | Water | Vents | 1 | 5 |
| ASV_0009_2008_05_26 | SRA009844.3 | -25.32 | 37.72 | 6 | 26/05/2008 | Water | Vents | 1 | 5 |
| ASV_0010_2001_08_04 | SRA009844.3 | -25.82 | 37.89 | 0.1 | 04/08/2001 | Water | Vents | 1 | 5 |
| ASV_0011_2001_07_20 | SRA009844.3 | -27.96 | 39.01 | 0.2 | 20/07/2001 | Water | Vents | 1 | 5 |
| ASV_0012_2001_07_16 | SRA009844.3 | -28.76 | 38.56 | 20 | 16/07/2001 | Water | Vents | 1 | 5 |
| ASV_0013_2001_08_10 | SRA009844.3 | -28.60 | 38.55 | 4 | 10/08/2001 | Water | Vents | 1 | 5 |
| ASV_0014_2007_07_16 | SRA009844.3 | -31.25 | 39.38 | 0.1 | 16/07/2007 | Water | Vents | 1 | 5 |
| AWP_0001_2007_08_23 | SRA009845.3 | -28.35 | 36.05 | 0 | 23/08/2007 | Water | Open Ocean | 1 | 5 |
| AWP_0003_2007_08_25 | SRA009845.3 | -32.12 | 34.21 | 0 | 25/08/2007 | Water | Open Ocean | 1 | 4 |
| AWP_0006_2007_08_25 | SRA009845.3 | -32.12 | 34.21 | 0 | 25/08/2007 | Water | Open Ocean | 1 | 4 |
| AWP_0007_2007_08_25 | SRA009845.3 | -32.43 | 35.48 | 0 | 25/08/2007 | Water | Open Ocean | 1 | 4 |
| AWP_0008_2007_08_26 | SRA009845.3 | -30.57 | 37.48 | 0 | 26/08/2007 | Water | Open Ocean | 1 | 5 |
| AWP_0009_2007_06_11 | SRA009845.3 | -18.88 | 37.34 | 3660 | 11/06/2007 | Water | Open Ocean | 1 | 5 |
| AWP_0010_2007_06_11 | SRA009845.3 | -18.88 | 37.34 | 2100 | 11/06/2007 | Water | Open Ocean | 1 | 5 |
| AWP_0011_2007_06_11 | SRA009845.3 | -18.88 | 37.34 | 1200 | 11/06/2007 | Water | Open Ocean | 1 | 5 |
| AWP_0012_2007_06_11 | SRA009845.3 | -18.88 | 37.34 | 800 | 11/06/2007 | Water | Open Ocean | 1 | 5 |
| AWP_0013_2007_06_11 | SRA009845.3 | -18.88 | 37.34 | 100 | 11/06/2007 | Water | Open Ocean | 1 | 5 |
| AWP_0014_2007_06_11 | SRA009845.3 | -18.88 | 37.34 | 0 | 11/06/2007 | Water | Open Ocean | 1 | 5 |
| AWP_0015_2007_06_02 | SRA009845.3 | -27.88 | 31.86 | 800 | 02/06/2007 | Water | Open Ocean | 1 | 4 |
| AWP_0016_2007_06_02 | SRA009845.3 | -27.88 | 31.86 | 100 | 02/06/2007 | Water | Open Ocean | 1 | 4 |
| BMO_0001_2007_09_20 | SRA009846.1 | 2.80 | 41.67 | 0.5 | 20/09/2007 | Water | Coastal | 2 | 4 |
| BMO_0002_2007_09_20 | SRA009846.1 | 2.80 | 41.65 | 5 | 20/09/2007 | Water | Coastal | 2 | 4 |
| BMO_0003_2007_09_21 | SRA009846.1 | 2.80 | 41.40 | 5 | 21/09/2007 | Water | Coastal | 2 | 4 |
| BMO_0004_2007_09_21 | SRA009846.1 | 2.82 | 41.15 | 5 | 21/09/2007 | Water | Open Ocean | 2 | 4 |
| BMO_0005_2007_09_22 | SRA009846.1 | 2.85 | 40.91 | 4 | 22/09/2007 | Water | Open Ocean | 2 | 4 |
| BMO_0006_2007_09_23 | SRA009846.1 | 2.85 | 40.65 | 5 | 23/09/2007 | Water | Open Ocean | 2 | 4 |
| BMO_0007_2007_09_23 | SRA009846.1 | 2.85 | 40.65 | 25 | 23/09/2007 | Water | Open Ocean | 2 | 4 |
| BMO_0008_2007_09_23 | SRA009846.1 | 2.85 | 40.65 | 65 | 23/09/2007 | Water | Open Ocean | 2 | 4 |
| BMO_0009_2007_09_22 | SRA009846.1 | 2.86 | 40.66 | 500 | 22/09/2007 | Water | Open Ocean | 2 | 4 |
| BMO_0010_2007_09_22 | SRA009846.1 | 2.86 | 40.66 | 2000 | 22/09/2007 | Water | Open Ocean | 2 | 4 |
| BMO_0011_2007_09_22 | SRA009846.1 | 2.80 | 41.40 | 500 | 22/09/2007 | Water | Coastal | 2 | 4 |
| BMO_0012_2007_09_21 | SRA009846.1 | 2.80 | 41.40 | 44 | 21/09/2007 | Water | Coastal | 2 | 4 |
| BMO_0013_2007_09_21 | SRA009846.1 | 2.86 | 40.66 | 44 | 21/09/2007 | Water | Open Ocean | 2 | 4 |
| BMO_0014_2007_09_23 | SRA009846.1 | 2.85 | 40.65 | 65 | 23/09/2007 | Water | Open Ocean | 2 | 4 |
| BMO_0015_2007_09_23 | SRA009846.1 | 2.85 | 40.65 | 5 | 23/09/2007 | Water | Open Ocean | 2 | 4 |
| BMO_0016_2007_09_20 | SRA009846.1 | 2.80 | 41.65 | 5 | 20/09/2007 | Water | Coastal | 2 | 4 |
| BSP_0001_2003_05_08 | SRA009836.1 | 18.24 | 58.60 | 4 | 08/05/2003 | Water | Coastal | 5 | 5 |
| BSP_0003_2003_06_18 | SRA009836.1 | 18.24 | 58.60 | 4 | 18/06/2003 | Water | Coastal | 5 | 5 |
| BSP_0004_2003_07_16 | SRA009836.1 | 18.24 | 58.60 | 4 | 16/07/2003 | Water | Coastal | 5 | 5 |
| BSP_0005_2003_07_30 | SRA009836.1 | 18.24 | 58.60 | 4 | 30/07/2003 | Water | Coastal | 5 | 5 |
| BSP_0006_2003_10_08 | SRA009836.1 | 18.24 | 58.60 | 4 | 08/10/2003 | Water | Coastal | 5 | 5 |
| BSP_0007_2004_05_17 | SRA009836.1 | 18.24 | 58.60 | 4 | 17/05/2004 | Water | Coastal | 5 | 5 |
| BSP_0009_2003_06_04 | SRA009836.1 | 18.24 | 58.60 | 4 | 04/06/2003 | Water | Coastal | 5 | 5 |
| BSP_0010_2003_08_27 | SRA009836.1 | 18.24 | 58.60 | 4 | 27/08/2003 | Water | Coastal | 5 | 5 |
| BSR_0001_2005_03_29 | SRA009847.1 | 30.75 | 42.50 | 66 | 29/03/2005 | Water | Anoxic | 2 | 4 |
| BSR_0002_2005_03_29 | SRA009847.1 | 30.75 | 42.50 | 85 | 29/03/2005 | Water | Anoxic | 2 | 4 |
| BSR_0003_2005_03_29 | SRA009847.1 | 30.75 | 42.50 | 105 | 29/03/2005 | Water | Anoxic | 2 | 4 |
| CAM_0001_2002_01_17 | SRA009848.1 | -64.05 | -64.77 | 3 | 17/01/2002 | Water | Coastal | 3 | 3 |
| CAM_0002_2002_07_17 | SRA009848.1 | -64.05 | -64.77 | 3 | 17/07/2002 | Water | Coastal | 3 | 3 |
| CAM_0003_2002_08_20 | SRA009848.1 | -64.05 | -64.77 | 3 | 20/08/2002 | Water | Coastal | 3 | 3 |
| CAM_0004_2006_02_28 | SRA009848.1 | -64.07 | -64.77 | 3 | 28/02/2006 | Water | Coastal | 3 | 3 |
| CAM_0005_2007_07_23 | SRA009848.1 | -68.42 | -50.67 | 3 | 23/07/2007 | Water | Coastal | 4 | 4 |
| CAM_0006_2007_10_09 | SRA009848.1 | -68.42 | -50.67 | 3 | 09/10/2007 | Water | Coastal | 4 | 4 |
| CAM_0007_2007_02_07 | SRA009848.1 | -68.42 | -50.67 | 3 | 07/02/2007 | Water | Coastal | 4 | 4 |
| CAM_0008_2007_05_04 | SRA009848.1 | -68.42 | -50.67 | 3 | 04/05/2007 | Water | Coastal | 4 | 4 |
| CAM_0009_2000_03_23 | SRA009848.1 | -2.89 | -55.13 | 11 | 23/03/2000 | Water | Open Ocean | 2 | 3 |
| CAM_0010_2000_03_25 | SRA009848.1 | -0.40 | -59.32 | 11 | 25/03/2000 | Water | Open Ocean | 2 | 3 |
| CAM_0011_2000_03_26 | SRA009848.1 | -5.47 | -67.02 | 11 | 26/03/2000 | Water | Open Ocean | 2 | 3 |
| CAM_0012_2000_03_29 | SRA009848.1 | -12.46 | -71.18 | 5 | 29/03/2000 | Water | Coastal | 3 | 3 |
| CAM_0013_2008_02_04 | SRA009848.1 | 175.56 | -59.40 | 10 | 04/02/2008 | Water | Open Ocean | 2 | 3 |
| CAM_0014_2008_02_06 | SRA009848.1 | 179.97 | -65.01 | 10 | 06/02/2008 | Water | Open Ocean | 2 | 1 |
| CAM_0015_2008_02_29 | SRA009848.1 | -179.09 | -69.43 | 5 | 29/02/2008 | Water | Open Ocean | 3 | 1 |
| CAM_0016_2008_02_13 | SRA009848.1 | 169.77 | -75.66 | 10 | 13/02/2008 | Water | Coastal | 3 | 1 |
| CAR_0001_1997_05_13 | SRA009849.1 | -64.67 | 10.50 | 260 | 13/05/1997 | Water | Anoxic | 5 | 4 |
| CAR_0002_2005_11_20 | SRA009849.1 | -64.67 | 10.50 | 330 | 20/11/2005 | Water | Anoxic | 5 | 4 |
| CAR_0003_1997_11_20 | SRA009849.1 | -64.67 | 10.50 | 350 | 20/11/1997 | Water | Anoxic | 5 | 4 |
| CAR_0004_2005_01_13 | SRA009849.1 | -64.67 | 10.50 | 320 | 13/01/2005 | Water | Anoxic | 5 | 4 |
| CAR_0005_2005_01_13 | SRA009849.1 | -64.67 | 10.50 | 250 | 13/01/2005 | Water | Anoxic | 5 | 4 |
| CAR_0006_2005_01_13 | SRA009849.1 | -64.67 | 10.50 | 290 | 13/01/2005 | Water | Anoxic | 5 | 4 |
| CAR_0007_2005_05_23 | SRA009849.1 | -65.58 | 10.67 | 320 | 23/05/2005 | Water | Anoxic | 5 | 4 |
| CAR_0008_2005_05_23 | SRA009849.1 | -64.67 | 10.50 | 300 | 23/05/2005 | Water | Anoxic | 5 | 4 |
| CAR_0009_2005_01_13 | SRA009849.1 | -65.58 | 10.67 | 245 | 13/01/2005 | Water | Anoxic | 5 | 4 |
| CAR_0010_2005_01_13 | SRA009849.1 | -65.58 | 10.67 | 280 | 13/01/2005 | Water | Anoxic | 5 | 4 |
| CAR_0011_2005_05_23 | SRA009849.1 | -64.67 | 10.50 | 260 | 23/05/2005 | Water | Anoxic | 5 | 4 |
| CAR_0012_2005_05_23 | SRA009849.1 | -64.67 | 10.50 | 340 | 23/05/2005 | Water | Anoxic | 5 | 4 |
| CAR_0013_2005_05_23 | SRA009849.1 | -64.67 | 10.50 | 500 | 23/05/2005 | Water | Anoxic | 5 | 4 |
| CAR_0015_2005_05_23 | SRA009849.1 | -65.58 | 10.67 | 290 | 23/05/2005 | Water | Anoxic | 5 | 4 |
| CFU_0001_2006_04_21 | SRA009850.1 | -9.51 | 35.56 | 3860 | 21/04/2006 | Sediment | Open Ocean | 5 | 4 |
| CFU_0003_2006_04_24 | SRA009850.1 | -7.33 | 35.66 | 1326 | 24/04/2006 | Sediment | Open Ocean | 5 | 4 |
| CFU_0005_2006_04_29 | SRA009850.1 | -7.33 | 35.66 | 1326 | 29/04/2006 | Sediment | Open Ocean | 5 | 4 |
| CFU_0007_2002_03_03 | SRA009850.1 | -78.08 | -11.06 | 262 | 03/03/2002 | Sediment | Open Ocean | 2 | 5 |
| CFU_0009_2002_06_01 | SRA009850.1 | 7.77 | 53.73 | 1 | 01/06/2002 | Sediment | Coastal | 5 | 5 |
| CFU_0010_2002_06_01 | SRA009850.1 | 7.73 | 53.72 | 1 | 01/06/2002 | Sediment | Coastal | 5 | 5 |
| CFU_0011_2006_10_25 | SRA009850.1 | 0.93 | 51.88 | 1 | 25/10/2006 | Sediment | Coastal | 5 | 5 |
| CNE_0001_2003_04_14 | SRA009852.1 | -70.80 | 42.71 | 0 | 14/04/2003 | Water | Coastal | 4 | 4 |
| CNE_0002_2003_07_03 | SRA009852.1 | -70.80 | 42.71 | 0 | 03/07/2003 | Water | Coastal | 4 | 4 |
| CNE_0003_2003_10_11 | SRA009852.1 | -70.80 | 42.71 | 0 | 11/10/2003 | Water | Coastal | 4 | 4 |
| CNE_0004_2004_01_22 | SRA009852.1 | -70.80 | 42.71 | 0 | 22/01/2004 | Water | Coastal | 4 | 4 |
| CRS_0001_2008_11_07 | SRA009853.1 | -157.80 | 21.47 | 0.5 | 07/11/2008 | Sediment | Coastal | 1 | 4 |
| CRS_0002_2008_11_07 | SRA009853.1 | -157.80 | 21.47 | 0.5 | 07/11/2008 | Sediment | Coastal | 1 | 4 |
| CRS_0003_2008_11_07 | SRA009853.1 | -157.80 | 21.47 | 0.5 | 07/11/2008 | Sediment | Coastal | 1 | 4 |
| CRS_0004_2008_11_07 | SRA009853.1 | -157.80 | 21.47 | 0.5 | 07/11/2008 | Sediment | Coastal | 1 | 4 |
| CRS_0009_2008_11_07 | SRA009853.1 | -157.80 | 21.47 | 0.5 | 07/11/2008 | Sediment | Coastal | 1 | 4 |
| CRS_0010_2008_11_07 | SRA009853.1 | -157.80 | 21.47 | 0.5 | 07/11/2008 | Sediment | Coastal | 1 | 4 |
| CRS_0011_2008_11_07 | SRA009853.1 | -157.80 | 21.47 | 0.5 | 07/11/2008 | Sediment | Coastal | 1 | 4 |
| CRS_0012_2008_11_07 | SRA009853.1 | -157.80 | 21.47 | 0.5 | 07/11/2008 | Sediment | Coastal | 1 | 4 |
| DAO_0001_2007_07_12 | SRA009854.1 | -61.76 | 68.83 | 1000 | 12/07/2007 | Water | Open Ocean | 5 | 4 |
| DAO_0003_2007_08_04 | SRA009854.1 | -150.23 | 71.96 | 1000 | 04/08/2007 | Water | Open Ocean | 5 | 1 |
| DAO_0004_2007_08_04 | SRA009854.1 | -150.23 | 71.96 | 395 | 04/08/2007 | Water | Open Ocean | 5 | 1 |
| DAO_0005_2007_08_12 | SRA009854.1 | -149.99 | 79.99 | 1000 | 12/08/2007 | Water | Open Ocean | 5 | 1 |
| DAO_0007_2007_08_12 | SRA009854.1 | -149.99 | 79.99 | 410 | 12/08/2007 | Water | Open Ocean | 5 | 1 |
| DAO_0009_2007_08_15 | SRA009854.1 | -140.19 | 77.00 | 1000 | 15/08/2007 | Water | Open Ocean | 5 | 1 |
| DAO_0010_2007_08_15 | SRA009854.1 | -140.19 | 77.00 | 400 | 15/08/2007 | Water | Open Ocean | 5 | 1 |
| DAO_0011_2007_08_20 | SRA009854.1 | -128.64 | 75.84 | 900 | 20/08/2007 | Water | Open Ocean | 5 | 1 |
| DAO_0012_2007_08_23 | SRA009854.1 | -140.09 | 73.97 | 1000 | 23/08/2007 | Water | Open Ocean | 5 | 1 |
| DAO_0013_2007_08_23 | SRA009854.1 | -140.09 | 73.97 | 428 | 23/08/2007 | Water | Open Ocean | 5 | 1 |
| DAO_0014_2007_09_18 | SRA009854.1 | 126.00 | 77.75 | 1000 | 18/09/2007 | Water | Open Ocean | 5 | 1 |
| DAO_0015_2007_09_21 | SRA009854.1 | 142.39 | 79.94 | 1200 | 21/09/2007 | Water | Open Ocean | 5 | 1 |
| DAO_0016_2007_09_21 | SRA009854.1 | 142.39 | 79.94 | 250 | 21/09/2007 | Water | Open Ocean | 5 | 1 |
| FIS_0001_2008_04_10 | SRA009856.7 | 8.40 | 55.04 | 0.05 | 10/04/2008 | Sediment | Coastal | 5 | 5 |
| FIS_0002_2008_04_10 | SRA009856.7 | 8.40 | 55.04 | 0.05 | 10/04/2008 | Sediment | Coastal | 5 | 5 |
| FIS_0003_2008_04_10 | SRA009856.7 | 8.40 | 55.04 | 0.125 | 10/04/2008 | Sediment | Coastal | 5 | 5 |
| FIS_0004_2008_04_10 | SRA009856.7 | 8.40 | 55.04 | 0.125 | 10/04/2008 | Sediment | Coastal | 5 | 5 |
| FIS_0005_2008_04_11 | SRA009856.7 | 8.41 | 55.04 | 0.05 | 11/04/2008 | Sediment | Coastal | 5 | 5 |
| FIS_0006_2008_04_11 | SRA009856.7 | 8.41 | 55.04 | 0.125 | 11/04/2008 | Sediment | Coastal | 5 | 5 |
| FIS_0007_2008_04_11 | SRA009856.7 | 8.41 | 55.04 | 0.05 | 11/04/2008 | Sediment | Coastal | 5 | 5 |
| FIS_0008_2008_04_11 | SRA009856.7 | 8.41 | 55.04 | 0.05 | 11/04/2008 | Sediment | Coastal | 5 | 5 |
| FIS_0009_2008_04_11 | SRA009856.7 | 8.41 | 55.04 | 0.05 | 11/04/2008 | Sediment | Coastal | 5 | 5 |
| FIS_0010_2008_04_11 | SRA009856.7 | 8.41 | 55.04 | 0.05 | 11/04/2008 | Water | Coastal | 5 | 5 |
| FIS_0011_2008_04_11 | SRA009856.7 | 8.41 | 55.04 | 0.125 | 11/04/2008 | Sediment | Coastal | 5 | 5 |
| FIS_0012_2008_04_11 | SRA009856.7 | 8.41 | 55.04 | 0.125 | 11/04/2008 | Sediment | Coastal | 5 | 5 |
| FIS_0013_2008_04_12 | SRA009856.7 | 8.43 | 55.03 | 0.05 | 12/04/2008 | Sediment | Coastal | 5 | 5 |
| FIS_0014_2008_04_12 | SRA009856.7 | 8.43 | 55.03 | 0.125 | 12/04/2008 | Sediment | Coastal | 5 | 5 |
| FIS_0015_2008_04_12 | SRA009856.7 | 8.44 | 55.03 | 0 | 12/04/2008 | Water | Coastal | 5 | 5 |
| FIS_0016_2008_04_12 | SRA009856.7 | 8.44 | 55.03 | 0 | 12/04/2008 | Water | Coastal | 5 | 5 |
| GMS_0001_2006_12_04 | SRA009857.4 | -77.12 | 34.74 | 1 | 04/12/2006 | Sediment | Coastal | 4 | 4 |
| GOA_0001_2006_09_05 | SRA009914.1 | 34.92 | 29.47 | 20 | 05/09/2006 | Water | Coastal | 5 | 4 |
| GOA_0002_2006_10_17 | SRA009914.1 | 34.92 | 29.47 | 20 | 17/10/2006 | Water | Coastal | 5 | 4 |
| GOA_0003_2006_11_19 | SRA009914.1 | 34.92 | 29.47 | 5 | 19/11/2006 | Water | Coastal | 5 | 4 |
| GOA_0004_2006_12_13 | SRA009914.1 | 34.92 | 29.47 | 5 | 13/12/2006 | Water | Coastal | 5 | 4 |
| GOA_0005_2007_01_15 | SRA009914.1 | 34.92 | 29.47 | 5 | 15/01/2007 | Water | Coastal | 5 | 4 |
| GOA_0006_2007_02_12 | SRA009914.1 | 34.92 | 29.47 | 5 | 12/02/2007 | Water | Coastal | 5 | 4 |
| GOA_0007_2007_03_15 | SRA009914.1 | 34.92 | 29.47 | 5 | 15/03/2007 | Water | Coastal | 5 | 4 |
| GOA_0008_2007_04_16 | SRA009914.1 | 34.92 | 29.47 | 5 | 16/04/2007 | Water | Coastal | 5 | 4 |
| GOA_0009_2007_05_14 | SRA009914.1 | 34.92 | 29.47 | 5 | 14/05/2007 | Water | Coastal | 5 | 4 |
| GOA_0010_2007_06_19 | SRA009914.1 | 34.92 | 29.47 | 5 | 19/06/2007 | Water | Coastal | 5 | 4 |
| GOA_0011_2007_09_18 | SRA009914.1 | 34.92 | 29.47 | 20 | 18/09/2007 | Water | Coastal | 5 | 4 |
| GOA_0012_2007_12_18 | SRA009914.1 | 34.92 | 29.47 | 5 | 18/12/2007 | Water | Coastal | 5 | 4 |
| GOA_0013_2007_07_24 | SRA009914.1 | 34.92 | 29.47 | 20 | 24/07/2007 | Water | Coastal | 5 | 4 |
| GOA_0014_2007_07_24 | SRA009914.1 | 34.92 | 29.47 | 100 | 24/07/2007 | Water | Coastal | 5 | 4 |
| GOA_0015_2007_07_24 | SRA009914.1 | 34.92 | 29.47 | 125 | 24/07/2007 | Water | Coastal | 5 | 4 |
| GOA_0016_2007_07_24 | SRA009914.1 | 34.92 | 29.47 | 700 | 24/07/2007 | Water | Coastal | 5 | 4 |
| HCW_0001_2007_04_04 | SRA009859.1 | -123.01 | 47.55 | 0 | 04/04/2007 | Water | Coastal | 3 | 4 |
| HCW_0002_2007_04_04 | SRA009859.1 | -123.01 | 47.55 | 145 | 04/04/2007 | Water | Coastal | 3 | 4 |
| HCW_0003_2007_04_06 | SRA009859.1 | -123.02 | 47.36 | 5 | 06/04/2007 | Water | Coastal | 3 | 4 |
| HCW_0004_2007_04_06 | SRA009859.1 | -123.02 | 47.36 | 50 | 06/04/2007 | Water | Coastal | 3 | 4 |
| HCW_0005_2007_06_11 | SRA009859.1 | -122.60 | 47.89 | 0 | 11/06/2007 | Water | Coastal | 3 | 4 |
| HCW_0006_2007_06_11 | SRA009859.1 | -122.60 | 47.89 | 124 | 11/06/2007 | Water | Coastal | 3 | 4 |
| HCW_0007_2007_06_13 | SRA009859.1 | -123.01 | 47.55 | 0 | 13/06/2007 | Water | Coastal | 3 | 4 |
| HCW_0008_2007_06_13 | SRA009859.1 | -123.01 | 47.55 | 144 | 13/06/2007 | Water | Coastal | 3 | 4 |
| HCW_0009_2007_06_13 | SRA009859.1 | -123.02 | 47.36 | 0 | 13/06/2007 | Water | Coastal | 3 | 4 |
| HCW_0010_2007_06_13 | SRA009859.1 | -123.02 | 47.36 | 40 | 13/06/2007 | Water | Coastal | 3 | 4 |
| HCW_0011_2007_06_13 | SRA009859.1 | -122.93 | 47.40 | 0 | 13/06/2007 | Water | Coastal | 3 | 4 |
| HCW_0012_2007_06_13 | SRA009859.1 | -122.93 | 47.40 | 11.7 | 13/06/2007 | Water | Coastal | 3 | 4 |
| HCW_0013_2007_10_23 | SRA009859.1 | -123.01 | 47.55 | 0 | 23/10/2007 | Water | Coastal | 3 | 4 |
| HCW_0014_2007_10_23 | SRA009859.1 | -123.01 | 47.55 | 140 | 23/10/2007 | Water | Coastal | 3 | 4 |
| HCW_0015_2007_10_23 | SRA009859.1 | -123.02 | 47.36 | 0 | 23/10/2007 | Water | Coastal | 3 | 4 |
| HCW_0016_2007_10_23 | SRA009859.1 | -123.02 | 47.36 | 50 | 23/10/2007 | Water | Coastal | 3 | 4 |
| ICR_0001_2007_05_17 | SRA009860.1 | 81.83 | 15.86 | 995 | 17/05/2007 | Sediment | Coastal | 3 | 4 |
| ICR_0002_2007_05_17 | SRA009860.1 | 81.83 | 15.86 | 995 | 17/05/2007 | Sediment | Coastal | 3 | 4 |
| ICR_0003_2008_09_05 | SRA009860.1 | 73.88 | 15.51 | 0 | 05/09/2008 | Sediment | Coastal | 3 | 4 |
| ICR_0004_2008_09_05 | SRA009860.1 | 73.80 | 15.65 | 0 | 05/09/2008 | Sediment | Coastal | 3 | 4 |
| ICR_0006_2005_04_15 | SRA009860.1 | 75.50 | -16.00 | 5000 | 15/04/2005 | Sediment | Open Ocean | 1 | 4 |
| ICR_0007_2007_05_06 | SRA009860.1 | 73.29 | 17.06 | 0 | 06/05/2007 | Sediment | Coastal | 3 | 4 |
| ICR_0008_2006_05_11 | SRA009860.1 | 73.28 | 17.06 | 0 | 11/05/2006 | Sediment | Coastal | 3 | 4 |
| ICR_0011_2004_11_05 | SRA009860.1 | 73.29 | 17.06 | 0 | 05/11/2004 | Sediment | Coastal | 3 | 4 |
| ICR_0012_2004_11_05 | SRA009860.1 | 73.29 | 17.06 | 0 | 05/11/2004 | Sediment | Coastal | 3 | 4 |
| ICR_0014_2007_05_17 | SRA009860.1 | 81.83 | 15.86 | 995 | 17/05/2007 | Sediment | Coastal | 3 | 4 |
| ICR_0015_2007_05_17 | SRA009860.1 | 73.90 | 15.12 | 0 | 17/05/2007 | Sediment | Coastal | 3 | 4 |
| ICR_0016_2007_05_17 | SRA009860.1 | 73.90 | 15.12 | 0 | 17/05/2007 | Sediment | Coastal | 3 | 4 |
| LCR_0001_2008_08_04 | SRA009862.3 | -67.05 | 17.94 | 2 | 04/08/2008 | Sediment | Coastal | 2 | 4 |
| LCR_0002_2009_01_13 | SRA009862.3 | -67.01 | 17.97 | 0.1 | 22/09/2008 | Water | Coastal | 2 | 4 |
| LCR_0003_2008_08_12 | SRA009862.3 | -67.04 | 17.88 | 50 | 12/08/2008 | Sediment | Coastal | 2 | 4 |
| LCR_0004_2008_09_22 | SRA009862.3 | -57.34 | -34.79 | 0.5 | 22/09/2008 | Water | Coastal | 3 | 4 |
| LCR_0006_2007_11_26 | SRA009862.3 | -50.29 | 0.71 | 0.1 | 26/11/2007 | Water | Coastal | 5 | 4 |
| LCR_0009_2008_04_25 | SRA009862.3 | -54.28 | -34.58 | 0.5 | 25/04/2008 | Water | Coastal | 3 | 4 |
| LCR_0010_2008_04_25 | SRA009862.3 | -54.27 | -34.67 | 0.6 | 25/04/2008 | Water | Coastal | 3 | 4 |
| LCR_0011_2006_06_29 | SRA009862.3 | -55.27 | -36.17 | 2 | 29/06/2006 | Water | Coastal | 3 | 4 |
| LCR_0013_2006_09_01 | SRA009862.3 | -57.68 | -38.69 | 5 | 01/09/2006 | Water | Coastal | 4 | 4 |
| LCR_0015_2008_05_06 | SRA009862.3 | -64.12 | -42.43 | 0 | 06/05/2008 | Sediment | Coastal | 4 | 4 |
| LCR_0016_2008_04_09 | SRA009862.3 | -67.37 | -45.75 | 0 | 09/04/2008 | Sediment | Coastal | 4 | 4 |
| LCY_0001_2003_05_11 | SRA009018.3 | -42.12 | 30.12 | 827 | 11/05/2003 | Sediment | Vents | 1 | 4 |
| LCY_0003_2003_05_04 | SRA009018.3 | -42.12 | 30.12 | 782 | 04/05/2003 | Sediment | Vents | 1 | 4 |
| LCY_0005_2003_05_16 | SRA009018.3 | -42.12 | 30.12 | 735 | 16/05/2003 | Sediment | Vents | 1 | 4 |
| LCY_0007_2003_05_04 | SRA009018.3 | -42.12 | 30.12 | 782 | 04/05/2003 | Sediment | Vents | 1 | 4 |
| MPI_0001_2004_05_06 | SRA009863.1 | 7.90 | 54.19 | 1 | 06/05/2004 | Water | Coastal | 5 | 5 |
| MPI_0002_2004_05_06 | SRA009863.1 | 7.90 | 54.19 | 1 | 06/05/2004 | Water | Coastal | 5 | 5 |
| MPI_0003_2005_05_31 | SRA009863.1 | 7.90 | 54.19 | 1 | 31/05/2005 | Water | Coastal | 5 | 5 |
| MPI_0004_2005_05_31 | SRA009863.1 | 7.90 | 54.19 | 1 | 31/05/2005 | Water | Coastal | 5 | 5 |
| MPI_0005_2007_03_05 | SRA009863.1 | 7.90 | 54.19 | 1 | 05/03/2007 | Water | Coastal | 5 | 5 |
| MPI_0006_2007_05_21 | SRA009863.1 | 7.90 | 54.19 | 1 | 21/05/2007 | Water | Coastal | 5 | 5 |
| MPI_0007_2007_05_21 | SRA009863.1 | 7.90 | 54.19 | 1 | 21/05/2007 | Water | Coastal | 5 | 5 |
| MPI_0008_2007_07_24 | SRA009863.1 | 7.90 | 54.19 | 1 | 24/07/2007 | Water | Coastal | 5 | 5 |
| MPI_0009_2004_05_17 | SRA009863.1 | 7.90 | 54.19 | 1 | 17/05/2004 | Water | Coastal | 5 | 5 |
| MPI_0010_2004_05_17 | SRA009863.1 | 7.90 | 54.19 | 1 | 17/05/2004 | Water | Coastal | 5 | 5 |
| MPI_0011_2004_05_24 | SRA009863.1 | 7.90 | 54.19 | 1 | 24/05/2004 | Water | Coastal | 5 | 5 |
| MPI_0012_2004_05_24 | SRA009863.1 | 7.90 | 54.19 | 1 | 24/05/2004 | Water | Coastal | 5 | 5 |
| MPI_0013_2005_05_17 | SRA009863.1 | 7.90 | 54.19 | 1 | 17/05/2005 | Water | Coastal | 5 | 5 |
| MPI_0014_2004_05_17 | SRA009863.1 | 7.90 | 54.19 | 1 | 17/05/2005 | Water | Coastal | 5 | 5 |
| MPI_0015_2004_05_24 | SRA009863.1 | 7.90 | 54.19 | 1 | 24/05/2005 | Water | Coastal | 5 | 5 |
| MPI_0016_2004_05_24 | SRA009863.1 | 7.90 | 54.19 | 1 | 24/05/2005 | Water | Coastal | 5 | 5 |
| NZS_0001_2007_04_09 | SRA009906.1 | 179.63 | -43.98 | 529 | 09/04/2007 | Sediment | Open Ocean | 3 | 3 |
| NZS_0002_2007_04_13 | SRA009906.1 | -175.55 | -43.29 | 644 | 13/04/2007 | Sediment | Open Ocean | 3 | 3 |
| NZS_0003_2007_04_16 | SRA009906.1 | -176.71 | -42.78 | 1025 | 16/04/2007 | Sediment | Open Ocean | 3 | 3 |
| NZS_0004_2007_04_26 | SRA009906.1 | 175.93 | -42.99 | 1197 | 26/04/2007 | Sediment | Open Ocean | 3 | 3 |
| NZS_0005_2007_04_24 | SRA009906.1 | 178.99 | -42.99 | 530 | 24/04/2007 | Sediment | Open Ocean | 3 | 3 |
| NZS_0006_2007_04_04 | SRA009906.1 | 178.64 | -44.13 | 516 | 04/04/2007 | Sediment | Open Ocean | 3 | 3 |
| NZS_0007_2007_04_06 | SRA009906.1 | 177.14 | -44.49 | 1241 | 06/04/2007 | Sediment | Open Ocean | 3 | 3 |
| NZS_0008_2007_04_05 | SRA009906.1 | 176.71 | -43.83 | 478 | 05/04/2007 | Sediment | Open Ocean | 3 | 3 |
| NZS_0009_2007_04_17 | SRA009906.1 | -178.62 | -43.52 | 424 | 17/04/2007 | Sediment | Open Ocean | 3 | 3 |
| NZS_0010_2007_04_07 | SRA009906.1 | 178.52 | -44.01 | 766 | 07/04/2007 | Sediment | Open Ocean | 3 | 3 |
| NZS_0011_2007_04_20 | SRA009906.1 | -178.34 | -42.53 | 1400 | 20/04/2007 | Sediment | Open Ocean | 3 | 3 |
| NZS_0012_2007_05_28 | SRA009906.1 | 167.53 | -38.62 | 482 | 28/05/2007 | Sediment | Open Ocean | 2 | 3 |
| NZS_0013_2007_05_30 | SRA009906.1 | 167.53 | -36.92 | 1217 | 30/05/2007 | Sediment | Open Ocean | 2 | 3 |
| NZS_0014_2007_06_07 | SRA009906.1 | 172.15 | -39.65 | 266 | 07/06/2007 | Sediment | Open Ocean | 1 | 3 |
| NZS_0015_2007_06_05 | SRA009906.1 | 170.85 | -40.87 | 544 | 05/06/2007 | Sediment | Open Ocean | 1 | 3 |
| NZS_0016_2007_06_04 | SRA009906.1 | 170.21 | -40.13 | 803 | 04/06/2007 | Sediment | Open Ocean | 1 | 3 |
| PML_0001_2007_02_16 | SRA009436.14 | -4.22 | 50.20 | 0 | 16/02/2007 | Water | Coastal | 5 | 5 |
| PML_0002_2007_03_07 | SRA009436.14 | -4.22 | 50.20 | 0 | 07/03/2007 | Water | Coastal | 5 | 5 |
| PML_0003_2007_03_26 | SRA009436.14 | -4.22 | 50.20 | 0 | 26/03/2007 | Water | Coastal | 5 | 5 |
| PML_0004_2007_04_23 | SRA009436.14 | -4.22 | 50.25 | 0 | 23/04/2007 | Water | Coastal | 5 | 5 |
| PML_0005_2007_05_08 | SRA009436.14 | -4.22 | 50.25 | 0 | 08/05/2007 | Water | Coastal | 5 | 5 |
| PML_0006_2007_06_04 | SRA009436.14 | -4.13 | 50.25 | 0 | 04/06/2007 | Water | Coastal | 5 | 5 |
| PML_0007_2007_06_25 | SRA009436.14 | -4.13 | 50.25 | 0 | 25/06/2007 | Water | Coastal | 5 | 5 |
| PML_0008_2007_07_30 | SRA009436.14 | -4.13 | 50.25 | 0 | 30/07/2007 | Water | Coastal | 5 | 5 |
| PML_0009_2007_08_20 | SRA009436.14 | -4.13 | 50.25 | 0 | 20/08/2007 | Water | Coastal | 5 | 5 |
| PML_0010_2007_09_29 | SRA009436.14 | -4.13 | 50.25 | 0 | 29/09/2007 | Water | Coastal | 5 | 5 |
| PML_0011_2007_10_25 | SRA009436.14 | -4.13 | 50.25 | 0 | 25/10/2007 | Water | Coastal | 5 | 5 |
| PML_0012_2007_12_12 | SRA009436.14 | -4.13 | 50.25 | 0 | 12/12/2007 | Water | Coastal | 5 | 5 |
| PML_0017_2008_02_20 | SRA009436.14 | -4.21 | 50.25 | 0 | 20/02/2008 | Water | Coastal | 5 | 5 |
| PML_0018_2008_03_05 | SRA009436.14 | -4.21 | 50.25 | 0 | 05/03/2008 | Water | Coastal | 5 | 5 |
| PML_0019_2008_03_17 | SRA009436.14 | -4.21 | 50.25 | 0 | 17/03/2008 | Water | Coastal | 5 | 5 |
| PML_0020_2008_04_21 | SRA009436.14 | -4.21 | 50.25 | 0 | 21/04/2008 | Water | Coastal | 5 | 5 |
| PML_0021_2008_05_06 | SRA009436.14 | -4.21 | 50.25 | 0 | 06/05/2008 | Water | Coastal | 5 | 5 |
| PML_0022_2008_06_02 | SRA009436.14 | -4.21 | 50.25 | 0 | 02/06/2008 | Water | Coastal | 5 | 5 |
| PML_0023_2008_06_23 | SRA009436.14 | -4.21 | 50.25 | 0 | 23/06/2008 | Water | Coastal | 5 | 5 |
| PML_0024_2008_07_21 | SRA009436.14 | -4.21 | 50.25 | 0 | 21/07/2008 | Water | Coastal | 5 | 5 |
| PML_0025_2008_08_20 | SRA009436.14 | -4.21 | 50.25 | 0 | 20/08/2008 | Water | Coastal | 5 | 5 |
| PML_0026_2008_09_22 | SRA009436.14 | -4.21 | 50.25 | 0 | 22/09/2008 | Water | Coastal | 5 | 5 |
| PML_0027_2008_10_27 | SRA009436.14 | -4.21 | 50.25 | 0 | 27/10/2008 | Water | Coastal | 5 | 5 |
| PML_0028_2008_12_08 | SRA009436.14 | -4.21 | 50.25 | 0 | 08/12/2008 | Water | Coastal | 5 | 5 |
| PML_0029_2008_01_28_Dp | SRA009436.14 | -4.21 | 50.25 | 0 | 28/01/2008 | Water | Coastal | 5 | 5 |
| PML_0030_2008_01_28_Np | SRA009436.14 | -4.24 | 50.26 | 0 | 28/01/2008 | Water | Coastal | 5 | 5 |
| PML_0031_2008_08_27_Dp | SRA009436.14 | -4.20 | 50.25 | 0 | 26/08/2008 | Water | Coastal | 5 | 5 |
| PML_0032_2008_08_27_Np | SRA009436.14 | -4.20 | 50.25 | 0 | 26/08/2008 | Water | Coastal | 5 | 5 |
| PML_0033_2008_08_28_Np | SRA009436.14 | -4.17 | 50.27 | 0 | 27/08/2008 | Water | Coastal | 5 | 5 |
| PML_0034_2008_08_28_Dp | SRA009436.14 | -4.15 | 50.27 | 0 | 27/08/2008 | Water | Coastal | 5 | 5 |
| PML_0035_2008_04_22_Dp | SRA009436.14 | -4.21 | 50.25 | 0 | 22/04/2008 | Water | Coastal | 5 | 5 |
| PML_0036_2008_04_22_Np | SRA009436.14 | -4.19 | 50.25 | 0 | 22/04/2008 | Water | Coastal | 5 | 5 |
| PML_0037_2008_04_22_D | SRA009436.14 | -4.21 | 50.25 | 0 | 22/04/2008 | Water | Coastal | 5 | 5 |
| PML_0038_2008_01_28_D | SRA009436.14 | -4.21 | 50.25 | 0 | 28/01/2008 | Water | Coastal | 5 | 5 |
| PML_39_2003_01_13 | SRA009436.14 | -4.21 | 50.25 | 0 | 13/01/2003 | Water | Coastal | 5 | 5 |
| PML_40_2003_03_31 | SRA009436.14 | -4.21 | 50.25 | 0 | 31/03/2003 | Water | Coastal | 5 | 5 |
| PML_41_2003_04_28 | SRA009436.14 | -4.21 | 50.25 | 0 | 28/04/2003 | Water | Coastal | 5 | 5 |
| PML_42_2003_05_26 | SRA009436.14 | -4.21 | 50.25 | 0 | 26/05/2003 | Water | Coastal | 5 | 5 |
| PML_43_2003_06_09 | SRA009436.14 | -4.21 | 50.25 | 0 | 09/06/2003 | Water | Coastal | 5 | 5 |
| PML_44_2003_07_07 | SRA009436.14 | -4.21 | 50.25 | 0 | 07/07/2003 | Water | Coastal | 5 | 5 |
| PML_45_2003_07_21 | SRA009436.14 | -4.21 | 50.25 | 0 | 21/07/2003 | Water | Coastal | 5 | 5 |
| PML_46_2003_08_04 | SRA009436.14 | -4.21 | 50.25 | 0 | 04/08/2003 | Water | Coastal | 5 | 5 |
| PML_47_2003_08_25 | SRA009436.14 | -4.21 | 50.25 | 0 | 25/08/2003 | Water | Coastal | 5 | 5 |
| PML_48_2003_09_29 | SRA009436.14 | -4.21 | 50.25 | 0 | 29/09/2003 | Water | Coastal | 5 | 5 |
| PML_49_2003_10_06 | SRA009436.14 | -4.21 | 50.25 | 0 | 06/10/2003 | Water | Coastal | 5 | 5 |
| PML_50_2003_12_08 | SRA009436.14 | -4.21 | 50.25 | 0 | 08/12/2003 | Water | Coastal | 5 | 5 |
| PML_51_2004_03_01 | SRA009436.14 | -4.21 | 50.25 | 0 | 01/03/2004 | Water | Coastal | 5 | 5 |
| PML_52_2004_03_15 | SRA009436.14 | -4.21 | 50.25 | 0 | 15/03/2004 | Water | Coastal | 5 | 5 |
| PML_53_2004_05_03 | SRA009436.14 | -4.21 | 50.25 | 0 | 03/05/2004 | Water | Coastal | 5 | 5 |
| PML_54_2004_05_31 | SRA009436.14 | -4.21 | 50.25 | 0 | 31/05/2004 | Water | Coastal | 5 | 5 |
| PML_55_2004_06_14 | SRA009436.14 | -4.21 | 50.25 | 0 | 14/06/2004 | Water | Coastal | 5 | 5 |
| PML_56_2004_06_21 | SRA009436.14 | -4.21 | 50.25 | 0 | 21/06/2004 | Water | Coastal | 5 | 5 |
| PML_57_2004_07_26 | SRA009436.14 | -4.21 | 50.25 | 0 | 26/07/2004 | Water | Coastal | 5 | 5 |
| PML_58_2004_08_23 | SRA009436.14 | -4.21 | 50.25 | 0 | 23/08/2004 | Water | Coastal | 5 | 5 |
| PML_59_2004_10_04 | SRA009436.14 | -4.21 | 50.25 | 0 | 04/10/2004 | Water | Coastal | 5 | 5 |
| PML_60_2004_10_25 | SRA009436.14 | -4.21 | 50.25 | 0 | 25/10/2004 | Water | Coastal | 5 | 5 |
| PML_61_2004_12_06 | SRA009436.14 | -4.21 | 50.25 | 0 | 06/12/2004 | Water | Coastal | 5 | 5 |
| PML_62_2004_12_20 | SRA009436.14 | -4.21 | 50.25 | 0 | 20/12/2004 | Water | Coastal | 5 | 5 |
| PML_63_2005_01_24 | SRA009436.14 | -4.21 | 50.25 | 0 | 24/01/2005 | Water | Coastal | 5 | 5 |
| PML_64_2005_02_28 | SRA009436.14 | -4.21 | 50.25 | 0 | 28/02/2005 | Water | Coastal | 5 | 5 |
| PML_65_2005_04_18 | SRA009436.14 | -4.21 | 50.25 | 0 | 18/04/2005 | Water | Coastal | 5 | 5 |
| PML_66_2005_05_04 | SRA009436.14 | -4.21 | 50.25 | 0 | 04/05/2005 | Water | Coastal | 5 | 5 |
| PML_67_2005_06_06 | SRA009436.14 | -4.21 | 50.25 | 0 | 06/06/2005 | Water | Coastal | 5 | 5 |
| PML_68_2005_06_20 | SRA009436.14 | -4.21 | 50.25 | 0 | 20/06/2005 | Water | Coastal | 5 | 5 |
| PML_69_2005_07_25 | SRA009436.14 | -4.21 | 50.25 | 0 | 25/07/2005 | Water | Coastal | 5 | 5 |
| PML_70_2005_08_15 | SRA009436.14 | -4.21 | 50.25 | 0 | 15/08/2005 | Water | Coastal | 5 | 5 |
| PML_71_2005_09_19 | SRA009436.14 | -4.21 | 50.25 | 0 | 19/09/2005 | Water | Coastal | 5 | 5 |
| PML_72_2005_10_17 | SRA009436.14 | -4.21 | 50.25 | 0 | 17/10/2005 | Water | Coastal | 5 | 5 |
| PML_73_2005_11_15 | SRA009436.14 | -4.21 | 50.25 | 0 | 15/11/2005 | Water | Coastal | 5 | 5 |
| PML_74_2005_12_05 | SRA009436.14 | -4.21 | 50.25 | 0 | 05/12/2005 | Water | Coastal | 5 | 5 |
| PML_75_2006_01_03 | SRA009436.14 | -4.21 | 50.25 | 0 | 03/01/2006 | Water | Coastal | 5 | 5 |
| PML_76_2006_03_06 | SRA009436.14 | -4.21 | 50.25 | 0 | 06/03/2006 | Water | Coastal | 5 | 5 |
| PML_77_2006_03_20 | SRA009436.14 | -4.21 | 50.25 | 0 | 20/03/2006 | Water | Coastal | 5 | 5 |
| PML_78_2006_04_11 | SRA009436.14 | -4.21 | 50.25 | 0 | 11/04/2006 | Water | Coastal | 5 | 5 |
| PML_79_2006_05_04 | SRA009436.14 | -4.21 | 50.25 | 0 | 04/05/2006 | Water | Coastal | 5 | 5 |
| PML_80_2006_06_06 | SRA009436.14 | -4.21 | 50.25 | 0 | 06/06/2006 | Water | Coastal | 5 | 5 |
| PML_81_2006_06_27 | SRA009436.14 | -4.21 | 50.25 | 0 | 27/06/2006 | Water | Coastal | 5 | 5 |
| PML_82_2006_07_17 | SRA009436.14 | -4.21 | 50.25 | 0 | 17/07/2006 | Water | Coastal | 5 | 5 |
| PML_83_2006_08_22 | SRA009436.14 | -4.21 | 50.25 | 0 | 22/08/2006 | Water | Coastal | 5 | 5 |
| PML_84_2006_09_12 | SRA009436.14 | -4.21 | 50.25 | 0 | 12/09/2006 | Water | Coastal | 5 | 5 |
| PML_85_2006_10_30 | SRA009436.14 | -4.21 | 50.25 | 0 | 30/10/2006 | Water | Coastal | 5 | 5 |
| PML_86_2006_12_12 | SRA009436.14 | -4.21 | 50.25 | 0 | 12/12/2006 | Water | Coastal | 5 | 5 |
| SMS_0001_2007_09_19 | SRA009865.1 | -123.02 | 35.16 | 3953.5 | 19/09/2007 | Sediment | Open Ocean | 3 | 4 |
| SMS_0002_2007_09_19 | SRA009865.1 | -123.02 | 35.16 | 3953.5 | 19/09/2007 | Sediment | Open Ocean | 3 | 4 |
| SMS_0003_2007_09_19 | SRA009865.1 | -123.02 | 35.16 | 3953.5 | 19/09/2007 | Sediment | Open Ocean | 3 | 4 |
| SMS_0004_2007_09_23 | SRA009865.1 | -123.02 | 35.16 | 3953.5 | 23/09/2007 | Sediment | Open Ocean | 3 | 4 |
| SMS_0005_2007_09_23 | SRA009865.1 | -123.02 | 35.16 | 3953.5 | 23/09/2007 | Sediment | Open Ocean | 3 | 4 |
| SMS_0006_2007_09_23 | SRA009865.1 | -123.02 | 35.16 | 3953.5 | 23/09/2007 | Sediment | Open Ocean | 3 | 4 |
| SMS_0007_2007_09_19 | SRA009865.1 | -123.02 | 35.16 | 3953.5 | 19/09/2007 | Sediment | Open Ocean | 3 | 4 |
| SMS_0008_2007_09_19 | SRA009865.1 | -123.02 | 35.16 | 3953.5 | 19/09/2007 | Sediment | Open Ocean | 3 | 4 |
| SMS_0009_2007_09_19 | SRA009865.1 | -123.02 | 35.16 | 3953.5 | 19/09/2007 | Sediment | Open Ocean | 3 | 4 |
| SMS_0010_2007_09_23 | SRA009865.1 | -123.02 | 35.16 | 3953.5 | 23/09/2007 | Sediment | Open Ocean | 3 | 4 |
| SMS_0011_2007_09_23 | SRA009865.1 | -123.02 | 35.16 | 3953.5 | 23/09/2007 | Sediment | Open Ocean | 3 | 4 |
| SMS_0012_2007_09_23 | SRA009865.1 | -123.02 | 35.16 | 3953.5 | 23/09/2007 | Sediment | Open Ocean | 3 | 4 |
| SMS_0013_2007_09_19 | SRA009865.1 | -123.02 | 35.16 | 3953.5 | 19/09/2007 | Sediment | Open Ocean | 3 | 4 |
| SMS_0014_2007_09_19 | SRA009865.1 | -123.02 | 35.15 | 3953.5 | 19/09/2007 | Sediment | Open Ocean | 3 | 4 |
| SMS_0015_2007_09_23 | SRA009865.1 | -123.02 | 35.16 | 3953.5 | 23/09/2007 | Sediment | Open Ocean | 3 | 4 |
| SMS_0016_2007_09_23 | SRA009865.1 | -123.02 | 35.16 | 3953.5 | 23/09/2007 | Sediment | Open Ocean | 3 | 4 |
| SSD_0001_2008_12_03 | SRA009867.1 | -70.00 | 42.00 | 0.01 | 03/12/2008 | Sediment | Coastal | 4 | 4 |
| SSD_0002_2008_12_03 | SRA009867.1 | -70.00 | 42.00 | 0.01 | 03/12/2008 | Sediment | Coastal | 4 | 4 |
| SSD_0003_2008_12_03 | SRA009867.1 | -70.00 | 42.00 | 0.01 | 03/12/2008 | Sediment | Coastal | 4 | 4 |
| SSD_0004_2008_12_03 | SRA009867.1 | -70.00 | 42.00 | 0.01 | 03/12/2008 | Sediment | Coastal | 4 | 4 |
| SSD_0005_2008_12_03 | SRA009867.1 | -70.00 | 42.00 | 0.01 | 03/12/2008 | Sediment | Coastal | 4 | 4 |
| SSD_0006_2008_12_03 | SRA009867.1 | -70.00 | 42.00 | 0.01 | 03/12/2008 | Sediment | Coastal | 4 | 4 |
| SSD_0007_2008_12_03 | SRA009867.1 | -70.00 | 42.00 | 0.01 | 03/12/2008 | Sediment | Coastal | 4 | 4 |
| SSD_0008_2008_12_03 | SRA009867.1 | -70.00 | 42.00 | 0.01 | 03/12/2008 | Sediment | Coastal | 4 | 4 |
| VAG_0001_2007_12_20 | SRA009868.2 | -73.07 | -36.69 | 15 | 20/12/2007 | Sediment | Coastal | 2 | 5 |
| VAG_0002_2007_12_20 | SRA009868.2 | -73.04 | -36.64 | 27 | 20/12/2007 | Sediment | Coastal | 2 | 5 |
| VAG_0003_2007_12_20 | SRA009868.2 | -73.00 | -36.60 | 35 | 20/12/2007 | Sediment | Coastal | 2 | 5 |
| VAG_0004_2008_01_08 | SRA009868.2 | -73.00 | -36.60 | 88 | 08/01/2008 | Sediment | Coastal | 2 | 5 |
| VAG_0005_2008_04_01 | SRA009868.2 | -73.07 | -36.69 | 15 | 01/04/2008 | Sediment | Coastal | 2 | 5 |
| VAG_0006_2008_04_01 | SRA009868.2 | -73.04 | -36.64 | 27 | 01/04/2008 | Sediment | Coastal | 2 | 5 |
| VAG_0007_2008_04_01 | SRA009868.2 | -73.00 | -36.60 | 35 | 01/04/2008 | Sediment | Coastal | 2 | 5 |
| VAG_0008_2008_04_21 | SRA009868.2 | -73.12 | -36.51 | 88 | 21/04/2008 | Sediment | Coastal | 2 | 5 |
| VAG_0009_2008_09_29 | SRA009868.2 | -73.07 | -36.69 | 15 | 29/09/2008 | Sediment | Coastal | 2 | 5 |
| VAG_0010_2008_09_29 | SRA009868.2 | -73.04 | -36.64 | 27 | 29/09/2008 | Sediment | Coastal | 2 | 5 |
| VAG_0011_2008_09_29 | SRA009868.2 | -73.00 | -36.60 | 35 | 29/09/2008 | Sediment | Coastal | 2 | 5 |
| VAG_0012_2008_09_23 | SRA009868.2 | -73.12 | -36.51 | 88 | 23/09/2008 | Sediment | Coastal | 2 | 5 |
| VAG_0013_2009_01_20 | SRA009868.2 | -73.07 | -36.69 | 15 | 20/01/2009 | Sediment | Coastal | 2 | 5 |
| VAG_0014_2009_01_20 | SRA009868.2 | -73.04 | -36.64 | 27 | 20/01/2009 | Sediment | Coastal | 2 | 5 |
| VAG_0015_2009_01_20 | SRA009868.2 | -73.00 | -36.60 | 35 | 20/01/2009 | Sediment | Coastal | 2 | 5 |
| VAG_0016_2009_01_15 | SRA009868.2 | -73.12 | -36.51 | 88 | 15/01/2009 | Sediment | Coastal | 2 | 5 |
| DSS_0001_1995_10_03 | NA | 60.27 | 16.05 | 4078 | 03/10/1995 | Sediment | Open Ocean | 4 | 4 |
| DSS_0002_1995_10_21 | NA | 65.03 | 10.05 | 4411 | 21/10/1995 | Sediment | Open Ocean | 1 | 4 |
| DSS_0003_1992_03_27 | NA | -19.58 | 47.17 | 4560 | 27/03/1992 | Sediment | Open Ocean | 2 | 5 |
| DSS_0004_1992_04_04 | NA | -19.58 | 47.17 | 4560 | 04/04/1992 | Sediment | Open Ocean | 2 | 5 |
| DSS_0005_1992_08_05 | NA | -19.58 | 47.17 | 4560 | 05/07/1992 | Sediment | Open Ocean | 2 | 5 |
| DSS_0006_1993_05_26 | NA | 25.86 | 34.74 | 1375 | 26/05/1993 | Sediment | Open Ocean | 2 | 4 |
| DSS_0007_1993_05_27 | NA | 26.10 | 34.41 | 4260 | 27/05/1993 | Sediment | Open Ocean | 2 | 4 |
| DSS_0008_1993_05_29 | NA | 28.57 | 33.60 | 2968 | 29/05/1993 | Sediment | Open Ocean | 2 | 4 |
| DSS_0009_1993_05_31 | NA | 30.60 | 32.68 | 1904 | 01/06/1993 | Sediment | Open Ocean | 2 | 4 |
| DSS_0010_1993_06_01 | NA | 31.89 | 32.01 | 193 | 05/06/1993 | Sediment | Coastal | 2 | 4 |
| DSS_0012_1993_09_01 | NA | 133.35 | 76.50 | 37 | 01/09/1993 | Sediment | Coastal | 5 | 1 |
| DSS_0013_1993_09_01 | NA | 133.35 | 76.50 | 37 | 01/09/1993 | Sediment | Coastal | 5 | 1 |
| DSS_0014_1993_09_05 | NA | 133.33 | 78.14 | 796 | 05/09/1993 | Sediment | Open Ocean | 5 | 1 |
| DSS_0015_1993_09_04 | NA | 133.19 | 78.39 | 2019 | 04/09/1993 | Sediment | Open Ocean | 5 | 1 |
| DSS_0016_1993_09_03 | NA | 130.60 | 79.65 | 3427 | 03/09/1993 | Sediment | Open Ocean | 5 | 1 |
| DSS_0017_1993_09_03 | NA | 130.60 | 79.65 | 3427 | 03/09/1993 | Sediment | Open Ocean | 5 | 1 |
| DSS_0018_1993_09_18 | NA | 114.46 | 77.52 | 65 | 18/09/1993 | Sediment | Coastal | 5 | 1 |
| DSS_0019_1993_09_17 | NA | 118.19 | 77.40 | 534 | 17/09/1993 | Sediment | Open Ocean | 5 | 1 |
| DSS_0020_1993_09_15 | NA | 118.58 | 77.68 | 1517 | 15/09/1993 | Sediment | Open Ocean | 5 | 1 |
| DSS_0021_1993_09_14 | NA | 118.74 | 78.67 | 2620 | 14/09/1993 | Sediment | Open Ocean | 5 | 1 |
| DSS_0022_2006_06_07 | NA | 143.89 | 39.11 | 5347 | 07/06/2006 | Sediment | Open Ocean | 2 | 5 |
| DSS_0023_2007_02_11 | NA | 177.02 | -40.02 | 1181 | 11/02/2007 | Sediment | Open Ocean | 3 | 3 |
| DSS_0024_2006_04_05 | NA | -93.83 | 21.33 | 2296 | 05/04/2006 | Sediment | Open Ocean | 2 | 4 |
| DSS_0025_2007_11_12 | NA | 30.35 | 32.53 | 1696 | 12/11/2007 | Sediment | Open Ocean | 2 | 4 |
| DSS_0026_2007_11_12 | NA | 30.35 | 32.53 | 1696 | 12/11/2007 | Sediment | Open Ocean | 2 | 4 |
| DSS_0027_2007_11_13 | NA | 30.36 | 32.53 | 1694 | 13/11/2007 | Sediment | Open Ocean | 2 | 4 |
| DSS_0028_2007_11_13 | NA | 30.36 | 32.53 | 1694 | 13/11/2007 | Sediment | Open Ocean | 2 | 4 |
| DSS_0029_2007_11_12 | NA | 30.35 | 32.53 | 1696 | 12/11/2007 | Sediment | Open Ocean | 2 | 4 |
| DSS_0030_2007_11_12 | NA | 30.35 | 32.53 | 1693 | 12/11/2007 | Sediment | Open Ocean | 2 | 4 |
| DSS_0031_2007_11_13 | NA | 30.36 | 32.53 | 1694 | 13/11/2007 | Sediment | Open Ocean | 2 | 4 |
| DSS_0032_2007_11_12 | NA | 30.35 | 32.53 | 1693 | 12/11/2007 | Sediment | Open Ocean | 2 | 4 |
| DSS_0033_2005_02_23 | NA | -14.00 | -70.00 | 4300 | 23/02/2005 | Sediment | Open Ocean | 3 | 3 |
| DSS_0034_2005_03_04 | NA | 7.35 | -28.11 | 5114 | 04/03/2005 | Sediment | Open Ocean | 1 | 4 |
| DSS_0035_2005_03_11 | NA | 0.90 | -9.93 | 1928 | 11/04/2005 | Sediment | Open Ocean | 2 | 4 |
| DSS_0036_2005_03_19 | NA | -5.58 | 0.83 | 5225 | 19/04/2005 | Sediment | Open Ocean | 2 | 4 |
| winter | NA | -70.67 | 41.53 | 0 | 15/01/2007 | Water | Coastal | 4 | 4 |
| HOT177_5 | SRA009870.3 | -158.00 | 22.75 | 5 | 26/01/2006 | Water | Open Ocean | 1 | 4 |
| HOT177_770 | SRA009870.3 | -158.00 | 22.75 | 770 | 27/01/2006 | Water | Open Ocean | 1 | 4 |
| HOT180_5 | SRA009870.3 | -158.00 | 22.75 | 5 | 02/04/2006 | Water | Open Ocean | 1 | 4 |
| HOT180_770 | SRA009870.3 | -158.00 | 22.75 | 770 | 02/04/2006 | Water | Open Ocean | 1 | 4 |
| HOT181_5 | SRA009870.3 | -158.00 | 22.75 | 5 | 27/05/2006 | Water | Open Ocean | 1 | 4 |
| HOT181_770 | SRA009870.3 | -158.00 | 22.75 | 770 | 27/05/2006 | Water | Open Ocean | 1 | 4 |
| HOT183_5 | SRA009870.3 | -158.00 | 22.75 | 5 | 13/07/2006 | Water | Open Ocean | 1 | 4 |
| HOT183_770 | SRA009870.3 | -158.00 | 22.75 | 770 | 13/07/2006 | Water | Open Ocean | 1 | 4 |
| HOT185_10 | SRA009870.3 | -158.00 | 22.75 | 10 | 17/09/2006 | Water | Open Ocean | 1 | 4 |
| HOT185_800 | SRA009870.3 | -158.00 | 22.75 | 800 | 17/09/2006 | Water | Open Ocean | 1 | 4 |
| HOT186_10 | SRA009870.3 | -158.00 | 22.75 | 10 | 22/10/2006 | Water | Open Ocean | 1 | 4 |
| HOT186_100 | SRA009870.3 | -158.00 | 22.75 | 100 | 22/10/2006 | Water | Open Ocean | 1 | 4 |
| HOT186_1000 | SRA009870.3 | -158.00 | 22.75 | 1000 | 22/10/2006 | Water | Open Ocean | 1 | 4 |
| HOT186_125 | SRA009870.3 | -158.00 | 22.75 | 125 | 22/10/2006 | Water | Open Ocean | 1 | 4 |
| HOT186_175 | SRA009870.3 | -158.00 | 22.75 | 175 | 22/10/2006 | Water | Open Ocean | 1 | 4 |
| HOT186_2000 | SRA009870.3 | -158.00 | 22.75 | 2000 | 22/10/2006 | Water | Open Ocean | 1 | 4 |
| HOT186_3000 | SRA009870.3 | -158.00 | 22.75 | 3000 | 22/10/2006 | Water | Open Ocean | 1 | 4 |
| HOT186_400 | SRA009870.3 | -158.00 | 22.75 | 400 | 22/10/2006 | Water | Open Ocean | 1 | 4 |
| HOT186_4000 | SRA009870.3 | -158.00 | 22.75 | 4000 | 22/10/2006 | Water | Open Ocean | 1 | 4 |
| HOT186_600 | SRA009870.3 | -158.00 | 22.75 | 600 | 22/10/2006 | Water | Open Ocean | 1 | 4 |
| HOT186_75 | SRA009870.3 | -158.00 | 22.75 | 75 | 22/10/2006 | Water | Open Ocean | 1 | 4 |
| HOT186_800 | SRA009870.3 | -158.00 | 22.75 | 800 | 22/10/2006 | Water | Open Ocean | 1 | 4 |
| HOT188_10 | SRA009870.3 | -158.00 | 22.75 | 10 | 11/12/2006 | Water | Open Ocean | 1 | 4 |
| HOT188_800 | SRA009870.3 | -158.00 | 22.75 | 800 | 11/12/2006 | Water | Open Ocean | 1 | 4 |
| HOT189_10 | SRA009870.3 | -158.00 | 22.75 | 10 | 08/02/2007 | Water | Open Ocean | 1 | 4 |
| HOT189_100 | SRA009870.3 | -158.00 | 22.75 | 100 | 08/02/2007 | Water | Open Ocean | 1 | 4 |
| HOT189_1000 | SRA009870.3 | -158.00 | 22.75 | 1000 | 08/02/2007 | Water | Open Ocean | 1 | 4 |
| HOT189_125 | SRA009870.3 | -158.00 | 22.75 | 125 | 08/02/2007 | Water | Open Ocean | 1 | 4 |
| HOT189_175 | SRA009870.3 | -158.00 | 22.75 | 175 | 08/02/2007 | Water | Open Ocean | 1 | 4 |
| HOT189_2000 | SRA009870.3 | -158.00 | 22.75 | 2000 | 08/02/2007 | Water | Open Ocean | 1 | 4 |
| HOT189_400 | SRA009870.3 | -158.00 | 22.75 | 400 | 08/02/2007 | Water | Open Ocean | 1 | 4 |
| HOT189_600 | SRA009870.3 | -158.00 | 22.75 | 600 | 08/02/2007 | Water | Open Ocean | 1 | 4 |
| HOT189_75 | SRA009870.3 | -158.00 | 22.75 | 75 | 08/02/2007 | Water | Open Ocean | 1 | 4 |
| HOT189_800 | SRA009870.3 | -158.00 | 22.75 | 800 | 08/02/2007 | Water | Open Ocean | 1 | 4 |
| HOT190_5 | SRA009870.3 | -158.00 | 22.75 | 5 | 21/03/2007 | Water | Open Ocean | 1 | 4 |
| HOT190_770 | SRA009870.3 | -158.00 | 22.75 | 770 | 21/03/2007 | Water | Open Ocean | 1 | 4 |
| HOT191_5 | SRA009870.3 | -158.00 | 22.75 | 5 | 05/05/2007 | Water | Open Ocean | 1 | 4 |
| HOT191_770 | SRA009870.3 | -158.00 | 22.75 | 770 | 05/05/2007 | Water | Open Ocean | 1 | 4 |
| HOT193_5 | SRA009870.3 | -158.00 | 22.75 | 5 | 08/07/2007 | Water | Open Ocean | 1 | 4 |
| HOT193_770 | SRA009870.3 | -158.00 | 22.75 | 770 | 08/07/2007 | Water | Open Ocean | 1 | 4 |
| HOT194_10 | SRA009870.3 | -158.00 | 22.75 | 10 | 05/08/2007 | Water | Open Ocean | 1 | 4 |
| HOT194_100 | SRA009870.3 | -158.00 | 22.75 | 100 | 05/08/2007 | Water | Open Ocean | 1 | 4 |
| HOT194_1000 | SRA009870.3 | -158.00 | 22.75 | 1000 | 05/08/2007 | Water | Open Ocean | 1 | 4 |
| HOT194_125 | SRA009870.3 | -158.00 | 22.75 | 125 | 05/08/2007 | Water | Open Ocean | 1 | 4 |
| HOT194_175 | SRA009870.3 | -158.00 | 22.75 | 175 | 05/08/2007 | Water | Open Ocean | 1 | 4 |
| HOT194_400 | SRA009870.3 | -158.00 | 22.75 | 400 | 05/08/2007 | Water | Open Ocean | 1 | 4 |
| HOT194_600 | SRA009870.3 | -158.00 | 22.75 | 600 | 05/08/2007 | Water | Open Ocean | 1 | 4 |
| HOT194_75 | SRA009870.3 | -158.00 | 22.75 | 75 | 05/08/2007 | Water | Open Ocean | 1 | 4 |
| HOT194_800 | SRA009870.3 | -158.00 | 22.75 | 800 | 05/08/2007 | Water | Open Ocean | 1 | 4 |
| HOT196_5 | SRA009870.3 | -158.00 | 22.75 | 5 | 03/10/2007 | Water | Open Ocean | 1 | 4 |
| HOT196_770 | SRA009870.3 | -158.00 | 22.75 | 770 | 04/10/2007 | Water | Open Ocean | 1 | 4 |
| HOT197_10 | SRA009870.3 | -158.00 | 22.75 | 10 | 03/12/2007 | Water | Open Ocean | 1 | 4 |
| HOT197_800 | SRA009870.3 | -158.00 | 22.75 | 800 | 03/12/2007 | Water | Open Ocean | 1 | 4 |
| HOT200_10 | SRA009870.3 | -158.00 | 22.75 | 10 | 24/02/2008 | Water | Open Ocean | 1 | 4 |
| HOT200_800 | SRA009870.3 | -158.00 | 22.75 | 800 | 24/02/2008 | Water | Open Ocean | 1 | 4 |
| HOT201_5 | SRA009870.3 | -158.00 | 22.75 | 5 | 28/05/2008 | Water | Open Ocean | 1 | 4 |
| HOT201_770 | SRA009870.3 | -158.00 | 22.75 | 770 | 28/05/2008 | Water | Open Ocean | 1 | 4 |
| HOT203_5 | SRA009870.3 | -158.00 | 22.75 | 5 | 27/07/2008 | Water | Open Ocean | 1 | 4 |
| HOT203_770 | SRA009870.3 | -158.00 | 22.75 | 770 | 27/07/2008 | Water | Open Ocean | 1 | 4 |
| HOT205_5 | SRA009870.3 | -158.00 | 22.75 | 5 | 11/10/2008 | Water | Open Ocean | 1 | 4 |
| HOT205_770 | SRA009870.3 | -158.00 | 22.75 | 770 | 11/10/2008 | Water | Open Ocean | 1 | 4 |
| HOT208_5 | SRA009870.3 | -158.00 | 22.75 | 5 | 21/01/2009 | Water | Open Ocean | 1 | 4 |
| HOT208_770 | SRA009870.3 | -158.00 | 22.75 | 770 | 21/01/2009 | Water | Open Ocean | 1 | 4 |
| 112R | SRA009950.3 | -25.00 | 50.40 | 4121 | 22/09/2002 | Water | Open Ocean | 2 | 5 |
| 115R | SRA009950.3 | -25.00 | 50.40 | 550 | 22/09/2002 | Water | Open Ocean | 2 | 5 |
| 137 | SRA009950.3 | -38.52 | 60.90 | 1710 | 28/05/2003 | Water | Open Ocean | 4 | 5 |
| 138 | SRA009950.3 | -38.52 | 60.90 | 710 | 28/05/2003 | Water | Open Ocean | 4 | 5 |
| 53R | SRA009950.3 | -29.13 | 58.30 | 1400 | 16/09/2002 | Water | Open Ocean | 4 | 5 |
| 55R | SRA009950.3 | -29.13 | 58.30 | 500 | 16/09/2002 | Water | Open Ocean | 4 | 5 |
| FS312 | NA | -129.99 | 45.92 | 0 | 04/09/2003 | Water | Vents | 3 | 4 |
| FS396 | NA | -129.99 | 45.94 | 0 | 26/09/2004 | Water | Vents | 3 | 4 |

**Supporting Figures**


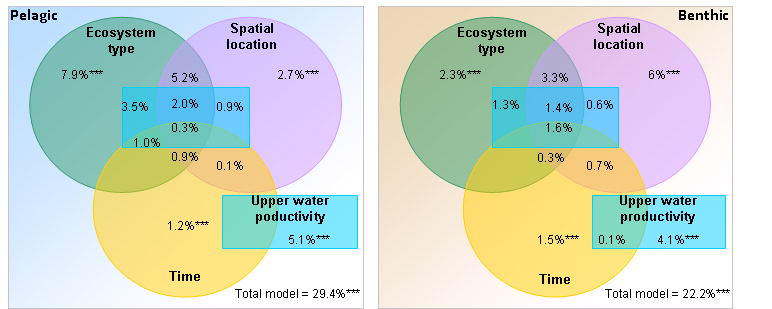


**Figure S1. Global beta-diversity patterns of marine bacterial communities within each realm.** The respective contribution of the ecosystem type, geographic location (transformed latitudes and longitudes, water depth), time (number of days since the first sampling) and upper water productivity (as defined by Longhurst’s primary production index and classes of capture fisheries yield) were assessed by using variation partitioning. The significance of each pure effect was validated by performing partial RDA with 1000 Monte Carlo permutation tests. Significance levels: *** *P*<0.001, ***P*<0.01, **P*<0.05. Covariation parts cannot be tested for significance because they are numerically deduced from the pure parts {Borcard, 1992 #70}
